# A formalization of Heisenbugs and their causes in terms of hyperproperties

**DOI:** 10.1007/s10270-025-01345-7

**Published:** 2026-02-24

**Authors:** Sarah Sallinger, Georg Weissenbacher, Florian Zuleger

**Affiliations:** TU Wien, Vienna, Austria

**Keywords:** Heisenbugs, Hyperproperties, Causal analysis, Counterfactuals

## Abstract

The already challenging task of identifying the cause of a bug becomes even more cumbersome if those bugs disappear or change their behavior under observation. Such bugs occur in a range of contexts including elusive concurrency bugs as well as unintended system alterations during debugging and—as a pun on the name of Werner Heisenberg—are often referred to as Heisenbugs. Heisenbugs can be caused by various sources of nondeterminism on different system levels, many of which developers and testers might not even be aware of. This paper provides formal foundations for rigorously reasoning about causes of Heisenbugs. It provides a formal definition of Heisenbugs in terms of a hyperproperty and introduces a framework for determining the causality of Heisenbugs in presence of multiple candidate causes based on said hyperproperty. We analyze the properties of causes and the implications on practical causal analyses. We provide two small case studies and a taxonomy of potential causes of Heisenbugs.

## Introduction

Bugs which change their behavior under observation are notoriously difficult to detect and fix. Inspired by Heisenberg’s uncertainty principle, such bugs are often referred to as *Heisenbugs*. Depending on the context, the term Heisenbug has been used to describe slightly different concepts. In the software engineering community, the term is used mostly for bugs whose analysis is hampered by the probe effect, i.e., an unintended alteration of the system behavior during debugging [25]. In the formal methods community, the term has been used to refer to elusive faults arising from executions that exhibit nondeterminism, in particular in the context of concurrent software [41]. In the context of automated testing, the term flaky test is used for inconsistently failing test cases [42], i.e., manifestations of Heisenbugs. As will become apparent in this paper, all the mentioned phenomena can be formalized in a uniform manner. In the rest of the paper, we hence use the term Heisenbug to refer to all the mentioned categories.^1^

*A Formalization of Heisenbugs* The first contribution of this paper is a formal definition of Heisenbugs (Sect. 2). The unifying characteristic of Heisenbugs in the above-mentioned categories is the existence of at least two system executions where one execution is correct and the other exhibits a bug. In terms of testing, the same test case sometimes succeeds and sometimes fails. We formalize this definition in terms of a hyperproperty [11], which checks for the existence of two terminating executions with equal inputs but deviating outcomes for a final assertion that is part of the system specification. Our definition accommodates deviations caused by nondeterminism in a single program, e.g., due to concurrency, as well as deviating behavior of different versions of the program, e.g., due to changes for debugging.

*Debugging Challenges* Previous studies show that Heisenbugs are prevalent even in mature software systems and that the bug fixing process takes significantly longer than for ordinary bugs [13]. Furthermore, Heisenbugs significantly complicate automated testing techniques, as they lead to flaky tests [42].

A major step in the debugging process is the identification of the bug’s root causes [50]. Developers reported this step to be particularly difficult for Heisenbugs [15] (referred to as flaky tests in this study). One reason for the complexity is that Heisenbugs can be caused by *mechanisms* (i.e., sources of nondeterminism or system alterations) located on all system levels ranging from the hardware level to the user program. The following examples illustrate some possible causes:

### Example 1

(*Concurrency*) We first present an example for a Heisenbug stemming from system-internal nondeterminism. The Therac-25 incident [34, 54], which resulted in the death of several cancer patients, is a notorious instance of an atomicity violation [38]. Listing 1.1 illustrates the problem, which is caused by the concurrent execution of two routines: the userInterface routine allows the operator to choose between high energy X-ray therapy (isXray) and a lower energy electron beam therapy (!isXray) and to set the intensity of the radiation (isHigh), providing inputs to the read procedure. The assume statement in line 6 prevents a selection of high-intensity electron therapy. The setup routine then processes these inputs to set the device parameters accordingly: a failed assertion represents the case where the patient is exposed to excessive radiation. Assume that the user changes the initial configuration from high-intensity X-ray treatment (isXray=true, isHigh=true) to low-energy electron therapy (isXray=false, isHigh=false) in lines 4 and 7. If a context switch occurs right after executing line 5, the assertion in line 13 will fail because isXray is already set to false and hence the filter is deactivated, while isHigh has not been updated yet, leading to a use of highEnergy. When userInterface is executed atomically, however, the assertion always holds.

**Figure:**
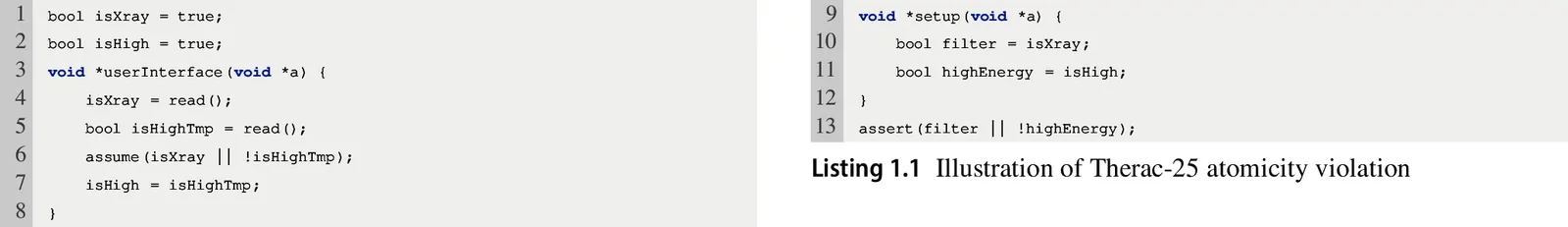

To sum up, there is a Heisenbug caused by different possible schedules, which is an example for the category of Heisenbugs arising from nondeterministic systems. Even if the scheduler might in fact be deterministic, its internal steps are not observable for the programmer (which we model using nondeterminism).

### Example 2

(*Floating Point Precision*) A prominent example for unintended system alterations are debugging statements that inadvertently change program outcomes. Consider Listing 1.2 (following [39]), which computes the square of $10^{308}$ and is expected to cause an overflow given the double-precision floating point representation. When compiled with optimization level-O3 and executed using x87 instructions, however, the computation results in $10^{308}$ rather than in an overflow, and the assertion fails. The reason is that the computation uses 80-bit floating point registers and performs rounding only once values are stored in 64-bit memory cells. Adding the printf statement in line 4 enforces such a write to memory, thus yielding the expected overflow, and the assertion holds.

**Figure:**
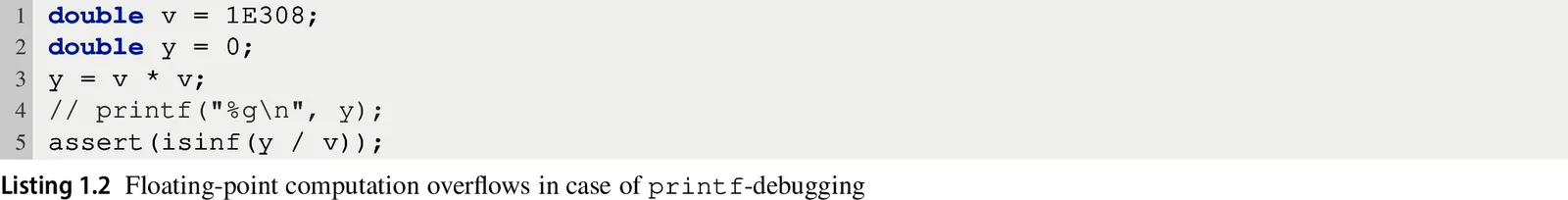

The failing and correct executions actually stem from two different system versions. In the considered execution model, the debugging statement changes the semantics of the program, introducing a probe effect which causes the Heisenbug.

*Multiple Causes* While the mechanisms causing the Heisenbugs in Examples 1 and 2 can still be easily identified, such an analysis becomes more challenging for more complex systems where several such mechanisms interact in a nontrivial manner [46] (as in Example 3 below).

### Example 3

(*Weak Memory Models*) Listing 1.3 shows Peterson’s mutual exclusion algorithm for two processes. Computer architectures with weak memory models relax the guarantees on the order in which variable assignments are observed across processor cores, causing the algorithm to fail. In particular, both processes might be in the critical section at the same time, violating the requirement for mutual exclusion, if they set their flags in lines 5 and 15 but do not commit the modifications from cache to shared memory before lines 8 and 18 are executed. Such an interleaving will not happen if writes are directly propagated to shared memory, resulting in a Heisenbug (see [49]). The reordering, however, is effectively prevented if there are printf statements in lines 7 and 17 (as might be the case during development) because the IO operation enforces a memory barrier. Hence, the bug only occurs once the printf statements are removed. Yet, the printf statements are not causally related to the Heisenbug (unlike in Example 2), as we will formally argue in Sect. 3.

**Figure:**
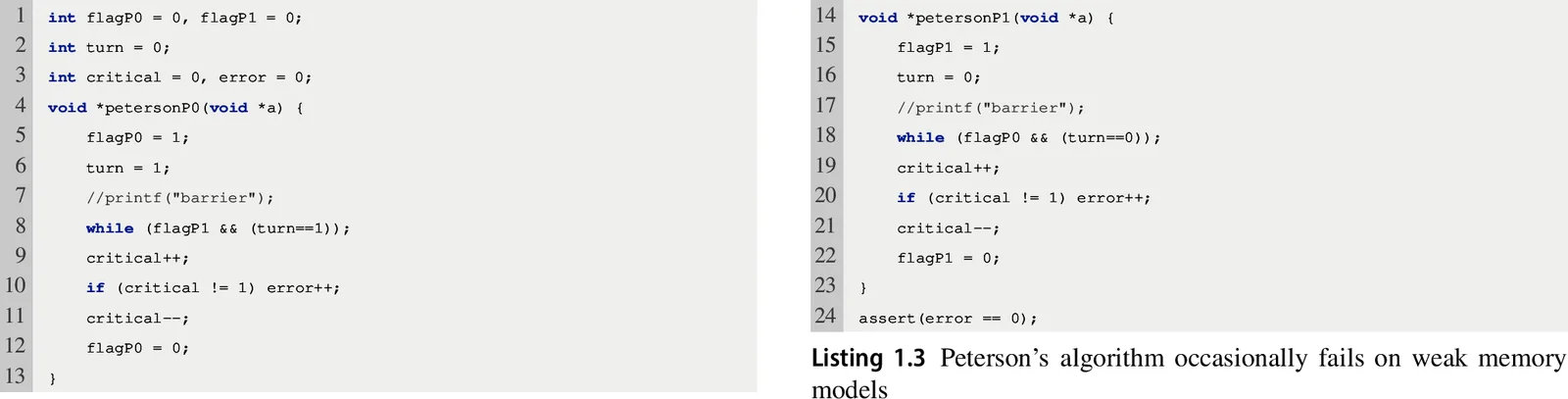

*Formal Causality Framework* In order to rigorously determine which mechanisms cause a Heisenbug in settings with multiple candidate causes, we present a formal causality definition based on Lewis’ counterfactuals [36] and the causality framework of Galles and Pearl [22] in Sect. 3. In counterfactual reasoning, an event is a cause of an effect, if in an alternative world where the cause does not occur, the effect does also not occur. In a nutshell, in a setting with multiple candidate mechanisms, a subset of the mechanisms is a cause of a Heisenbug if there are correct as well as failing executions which agree on the behavior of all other given mechanisms. This is formalized by means of a hyperproperty resembling our formal definition of Heisenbugs.

Note that our formal definition of causes refers to alternative scenarios for counterfactual reasoning. This requires the sources of nondeterminism to be made explicit in the underlying model (or controllable in the system under test, respectively). In practice, however, identifying and controlling all possible sources of nondeterminism is hardly feasible. Therefore, we show that our causal analysis yields a sound underapproximation of a cause even if some sources of nondeterminism remain unknown or uncontrollable (see Sect. 3.3).

*Taxonomy of Common Causes* Despite our formal guarantees for nondeterministic systems, an exact causal analysis is only possible if all potential causes are known; i.e., the alternative worlds can only be considered if all nondeterministic mechanisms are made explicit in the model. The more nondeterministic mechanisms are considered in a causal analysis, the more complete is its result. In order to raise awareness for sources of nondeterminism that practical analyses could take into account, Sect. 5 presents a taxonomy of common Heisenbug causes arising at different system levels and with varying degrees of observability and controllability.

*Timing of Inputs and Quiescence* In addition to the sources of nondeterminism discussed in our taxonomy, the arrival time of inputs (or their absence) may subtly influence the behavior of a system. The user, however, may be oblivious to the length of periods of quiescence in the input sequence. Section 4 discusses the consequences of such (potentially unobservable) deviations.

*Methodology* Based on the results outlined above, we present an iterative refinement methodology for causal analysis and discuss practical challenges. We showcase how the methodology can be applied for analyses based on model checking and testing (Sect. 6).

*General and Actual Causality* While our formalization of causes is more closely related to work of Galles and Pearl [22], one of the most prominent representatives of counterfactual based causal frameworks is Halpern and Pearl’s *actual causality* [26, 27]. Section 7.1 highlights the differences and similarities between our notion of causality and actual causality.

*Main Contributions* We summarize the contributions of our paper below. Contributions that are new compared to the conference version of this article [48] are marked with ⋆.

- A formal definition of Heisenbugs in reactive systems in terms of a hyperproperty, in the presence of system-internal nondeterminism and/or unintended system alternations (Sect. 2).
- A hyperproperty-based definition of the causality of Heisenbugs in the presence of several potential causes and nondeterminism (see Sect. 3).

- ⋆ A discussion of (unobservable) quiescence in the input sequence (Sect. 4).
- ⋆ A taxonomy of sources of nondeterminism which can form the basis as well as improve the accuracy of future causal analyses based on our framework (Sect. 5).

- A methodology for causal analysis based on iterative refinement, illustrated for software testing as well as model checking (see Sect. 6).
  - ⋆ The description of the test-based approach has been extended significantly with additional technical material on the sequentialization of concurrent programs.

- ⋆ A detailed discussion of the relationship between the introduced notion of causality and Halpern and Pearl’s *actual causality* (Sect. 7.1).

**Fig. 1:**
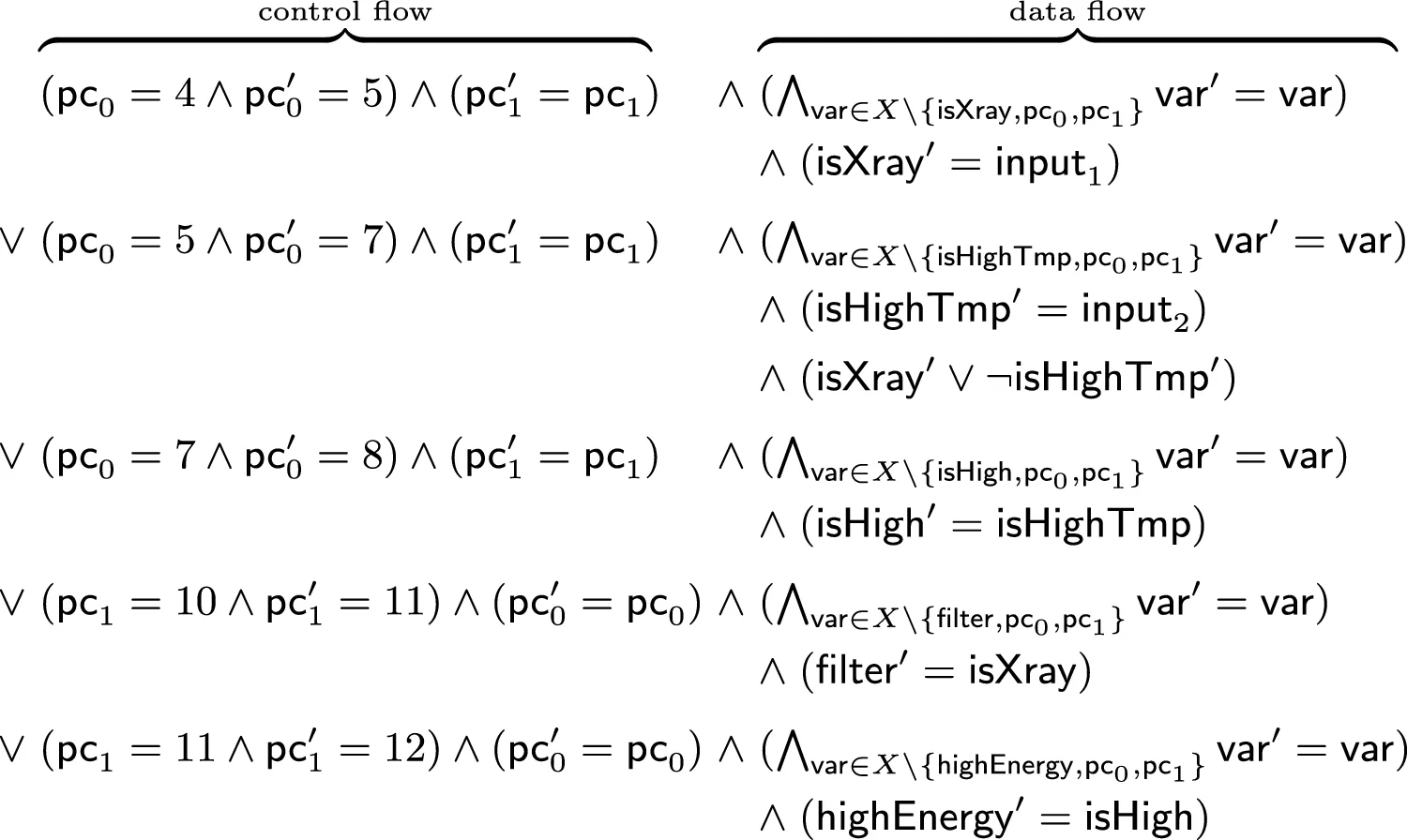
Transition relation for Listing 1.1

## A formalization of Heisenbugs

This section provides our system model and a formal definition of Heisenbugs.

### System model

In the following, the term *formula* refers to a first-order formula with a background theory that fixes the interpretations of predicates and function symbols.

#### Definition 1

- A **Symbolic Transition System** (STS) is a tuple $(X, I, \textsf{init}, \textsf{final}, T)$, where:
  - the **initial condition** $\textsf{init}$ is a formula over X⊎I, where *X* and *I* are some disjoint sets of **system** and **input variables**,
  - the **final condition** $\textsf{final}$ is a formula over *X*, and
  - the **transition relation** T is a formula over $$X \uplus I \uplus X'$$, where the variables $X^{\prime }$ denote primed copies of the variables *X*.
- **Configurations and States** Let $\textsf{Val}$ be a domain of **values**. Following [55], we assume that $\textsf{Val}$ includes a special value τ that represents *quiescence*, i.e., the absence of an input. A **configuration** *c* of an STS is a mapping of the variables in (X⊎I) to values in $\textsf{Val}$; we write *c*.*v* for the value of a variable $v \in (X \uplus I)$ in configuration *c*. A **state** *s* is a mapping of the variables in *X* to values in $\textsf{Val}$. An **input** *i* is a mapping of the variables in *I* to values in $\textsf{Val}$. The state ${X}({c})$ (input ${I}({c})$, respectively) of a configuration *c* is the restriction of the mapping to variables in *X* (*I*, respectively).
- **Conditions and Transitions** For a formula φ and a mapping *m* of variables to values, we write $$m \models \varphi $$ if φ evaluates to true under *m*. A configuration *c* is **initial** if $$c \models \textsf{init} $$. A state *s* is **final** if $$s \models \textsf{final} $$. A configuration *c* is **final** if $${X}({c})$$ is final. A state $$s: X\rightarrow \textsf{Val} $$ is **successor state** of configuration *c* if $$\langle {c},{s'}\rangle \models T $$, where $$s': X'\rightarrow \textsf{Val} $$ is the function that maps each primed variable $x^{\prime }\in X^{\prime }$ to *s*(*x*) and $$\langle {\cdot },{\cdot }\rangle $$ denotes the union of two mappings with disjoint domains. We call $$c_{i+1}$$ a **successor configuration** of $$c_i$$ if $${X}({c_{i+1}})$$ is a successor state of $$c_i$$. We require that final configurations do not have successor configurations.
- **Traces and Executions** A (finite or infinite) **trace** π of an STS is a sequence of configurations $c_0,\ldots ,c_n$ where $c_0\models \textsf{init}$, and $c_{i+1}$ is a successor of $c_i$ for all i≥0. An **execution** of an STS is a finite trace $\pi {\mathop {=}\limits ^{\tiny def }}c_0,\ldots ,c_n$ such that $c_n$ is a final configuration. As for configurations, we write X(π) and I(π), respectively, for the projection of π to *X* and *I*, respectively. Analogously, we use v(π) for the projection of π to a variable $v \in (X \uplus I)$.

It is straightforward to represent programs such as the examples from the introduction as symbolic transition systems:

#### Example 4

Listing 1.1 can be modeled as an STS with $I = \{\textsf{input}_1,\textsf{input}_2\}$ modeling the user inputs provided to the read procedure and $X = \{\textsf{isXray},\textsf{isHigh},[1] \textsf{isHighTmp},[1] \textsf{filter},[1] \textsf{highEnergy}, \textsf{pc}_0, \textsf{pc}_1\}$, where the variables $\textsf{pc}_0$ and $\textsf{pc}_1$ model the program counters of the two threads. The initial condition is $(\textsf{isXray}\wedge \textsf{isHigh} \wedge \textsf{pc}_0 = 4 \wedge \textsf{pc}_1 = 10)$. The final condition is $(\textsf{pc}_0 = 8 \wedge \textsf{pc}_1 = 12)$ and describes that both traces have reached their final program location. The transition relation T shown in Fig. 1 is a disjunctive partitioning that represents a case split over all possible combinations of program locations, where the thread to be executed in each step is chosen nondeterministically.^2^

While Example 4 illustrates the case of nondeterminism in a single system version, we next exemplify how to model system alterations in our formal model: the original and the altered system can be combined in one $\text {STS}$ with an initial nondeterministic choice between two disjuncts of the transition relation (cf. [19]).

#### Example 5

The floating point program from Listing 1.2 can be modeled as an $\text {STS}$ where $X = \{\textsf{v}, \textsf{y}, \textsf{pc}, \textsf{print}\}$, I=∅, the initial condition is $(\textsf{pc} = 3 \wedge \textsf{v} = 10^{308} \wedge \textsf{y} = 0)$, the final condition is $\textsf{pc} = 5$, and the transition relation is defined as $(\textsf{pc} = 3 \wedge \textsf{pc}' = 5 \wedge \textsf{y}' = \textsf{v} * \textsf{v} \wedge \textsf{v}' = \textsf{v} \wedge \lnot \textsf{print} \wedge \textsf{print}' = \textsf{print}) \vee (\textsf{pc} = 3 \wedge \textsf{pc}' = 5 \wedge \textsf{y}' = \textsf{convert64}(\textsf{v} * \textsf{v}) \wedge \textsf{v}' = \textsf{v} \wedge \textsf{print} \wedge \textsf{print}' = \textsf{print})$ where $\textsf{convert64}$ is a function producing the 64-bit representation of the number. The initial condition does not constrain $\textsf{print}$, i.e., the initial value of $\textsf{print}$ can be arbitrary; this initial (nondeterministic) choice then fixes the respective disjunct of the transition relation depending on whether the printf-statement is present or not.

In the following, we formally define a number of useful properties of STSs.

#### Definition 2

(Termination) An STS is *terminating* if the STS does not have infinite traces.

#### Definition 3

(Input Determinism) An STS is *input-deterministic* if 1) for every input *i* there is at most one state *s* such that $\langle {s},{i}\rangle \models \textsf{init}$, and 2) for every state *s* and every input *i* there is at most one successor state. Otherwise, it is *nondeterministic*.

#### Definition 4

(Input-enabled) An STS is *input-enabled* if 1) for every input *i* there is at least one state *s* such that $\langle {s},{i}\rangle \models \textsf{init}$, and 2) every configuration that is not final has at least one successor. In case 2) is violated, we call the transition relation *partial*.

We next define assertions as well as succeeding and failing executions:

#### Definition 5

(Assertions, Succeeding and Failing Executions) An *assertion* is a formula φ over the system variables *X*. An execution $\pi {\mathop {=}\limits ^{\tiny def }}c_0,\ldots ,c_n$ *succeeds* with respect to φ if ${X}({c_n})\models \varphi$. Similarly, π *fails* if ${X}({c_n})\models \lnot \varphi$. Abusing our notation, we write $\pi \models \varphi$ if π succeeds and $\pi \not \models \varphi$ if π fails.

We note that without input-enabledness, which we do not require in general, traces can get stuck at nonfinal configurations: For example, in Fig. 1, any state with $\textsf{pc}_0=5,\textsf{pc}_1=12,\textsf{isXray}=\textsf{false},\textsf{input}_2=\textsf{true}$ does not have a successor. For such traces, it is not meaningful to argue whether they satisfy an assertion. This is why Definition 5 refers to executions, i.e., traces that end in a final configuration. Moreover, Definition 5 disregards infinite traces, as we limit ourselves in this paper to Heisenbugs that are observable in a finite amount of time; we leave the extension to nonterminating traces to future work.

#### Example 6

**Fig. 2:**
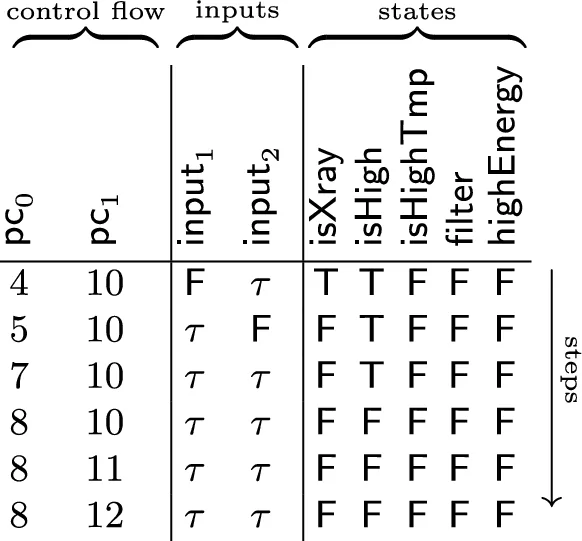
Execution $\pi _1$ of Fig. 1

**Fig. 3:**
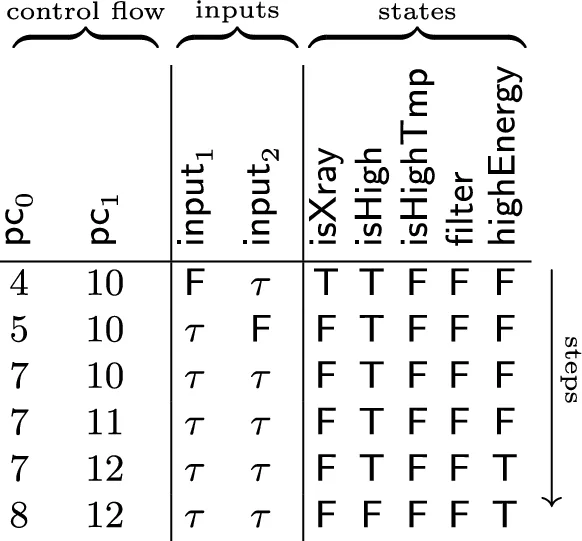
Execution $\pi _2$ of Fig. 1

Figures 2 and 3 show executions of the STS from Example 4. For $\varphi {\mathop {=}\limits ^{\tiny def }}(\textsf{filter}\vee \lnot \textsf{highEnergy})$ (the assertion in line 13), we have $\pi _1\models \varphi$ and $\pi _2\not \models \varphi$.

We presume that a system contains a bug if it has at least one failing execution, i.e., we assume that the assertion φ correctly encodes desired behavior of the program:^3^

#### Definition 6

(Bug) Let $(X, I, \textsf{init}, \textsf{final}, T)$ be an STS and the assertion φ be a formula over *X*. The STS contains a *bug* with respect to φ if there exists a failing counterexample execution:

$$\begin{aligned} \exists \pi _c\,.\, \pi _c \not \models \varphi . \end{aligned}$$

A violation of the property φ in Definition 6, however, does not necessarily constitute a Heisenbug.

### Formal definition of Heisenbugs

Heisenbugs are special bugs which occur only in some, but not in all executions. We express this in terms of a hyperproperty [11]. Unlike properties over single executions (such as Definition 6), hyperproperties relate sets of traces, allowing us to characterize Heisenbugs by juxtaposing the behavior of two executions. In particular, we require at least one succeeding and one failing execution induced by the same input (as deviating behavior is to be expected for differing inputs); we note that this implies that only nondeterministic STSs can have Heisenbugs.

#### Definition 7

(Heisenbug) An STS $(X, I, \textsf{init}, \textsf{final}, T)$ contains a *Heisenbug* with respect to an assertion φ if

$$\begin{aligned} \exists \pi _c, \pi _w\, .\, {I}({\pi _c})={I}({\pi _w})\wedge \pi _c \not \models \varphi \wedge \pi _w \models \varphi . \end{aligned}$$

The execution $\pi _c$ is the *counterexample execution*, $\pi _w$ is the *witness execution*.

We emphasize that the definition is expressed in terms of a hyperproperty stating that the inputs of the two traces must match. This condition cannot be expressed as a simple trace property. Moreover, we remark that Definition 7 is amenable to hyperproperty model checking and can be checked with any algorithm or verification tool that supports hypersafety properties (such as [4, 7, 20]).

#### Example 7

The Therac-25 example contains a Heisenbug with counterexample execution $\pi _2$ from Fig. 3 and witness execution $\pi _1$ from Fig. 2.

## Causality

In this section, we extend the hyper property from Definition 7 to counterfactually reason about the causality of Heisenbugs. We first present a refinement step for making potential causes explicit in the model and then introduce formal definitions of causality in deterministic as well as nondeterministic systems.

### Modeling sources of nondeterminism

Nondeterminism in a system may be the result of partial observability or incomplete modeling. In such cases, the nondeterminism can be eliminated by refining the model with the relevant information. In general, every source of nondeterminism can—at least in principle—be accounted for by means of prophecy variables [1]. For the purpose of causality analysis, we require the sources of nondeterminism (which we call *mechanisms*) to be made explicit.

To formalize this idea, we introduce refinements of a transition system:

#### Definition 8

(Refinement) Let $S=(X, I, \textsf{init}, \textsf{final}, T)$ be an STS. We say an STS ${S}_{\textsf{ref}} = (X \uplus {X}_{\textsf{ref}}, I \uplus {I}_{\textsf{ref}}, {\textsf{init}}_{\textsf{ref}}, {\textsf{final}}_{\textsf{ref}}, {T}_{\textsf{ref}})$ is a *refinement* of *S* iff

1. for all $s: X\rightarrow \textsf{Val}$, $i: I\rightarrow \textsf{Val}$, $s': X'\rightarrow \textsf{Val}$, ${s}_{\textsf{ref}}: {X}_{\textsf{ref}}\rightarrow \textsf{Val}$, ${i}_{\textsf{ref}}: {I}_{\textsf{ref}}\rightarrow \textsf{Val}$, and ${s}_{\textsf{ref}}': {X}_{\textsf{ref}}'\rightarrow \textsf{Val}$ such that $\langle {\langle }{\langle {s},{{s}_{\textsf{ref}}}\rangle },{\langle {i},{{i}_{\textsf{ref}}}\rangle }{\rangle },{\langle {s'},{{s}_{\textsf{ref}}'}\rangle }\rangle \models {T}_{\textsf{ref}}$ we have that $\langle {\langle }{s},{i}{\rangle },{s'}\rangle \models T$, and for all $s: X\rightarrow \textsf{Val}$, $i: I\rightarrow \textsf{Val}$, ${s}_{\textsf{ref}}: {X}_{\textsf{ref}}\rightarrow \textsf{Val}$ such that $\langle {\langle }{s},{i}{\rangle },{s'}\rangle \models T$ there are mappings ${i}_{\textsf{ref}}: {I}_{\textsf{ref}}\rightarrow \textsf{Val}$, ${s}_{\textsf{ref}}': {X}_{\textsf{ref}}'\rightarrow \textsf{Val}$ such that $\langle {\langle }{\langle {s},{{s}_{\textsf{ref}}}\rangle },{\langle {i},{{i}_{\textsf{ref}}}\rangle }{\rangle },{\langle {s'},{{s}_{\textsf{ref}}'}\rangle }\rangle \models {T}_{\textsf{ref}}$,
2. for all $s: X\rightarrow \textsf{Val}$, $i: I\rightarrow \textsf{Val}$, ${s}_{\textsf{ref}}: {X}_{\textsf{ref}}\rightarrow \textsf{Val}$, ${i}_{\textsf{ref}}: {I}_{\textsf{ref}}\rightarrow \textsf{Val}$ such that $\langle {\langle {s},{{s}_{\textsf{ref}}}\rangle },{\langle {i},{{i}_{\textsf{ref}}}\rangle }\rangle \models {\textsf{init}}_{\textsf{ref}}$ we have $\langle {s},{i}\rangle \models \textsf{init}$, and for all $s: X\rightarrow \textsf{Val}$ and $i: I\rightarrow \textsf{Val}$ such that $\langle {s},{i}\rangle \models \textsf{init}$ there are mappings ${i}_{\textsf{ref}}: {I}_{\textsf{ref}}\rightarrow \textsf{Val}$ and ${s}_{\textsf{ref}}: {X}_{\textsf{ref}}\rightarrow \textsf{Val}$ such that $\langle {\langle {s},{{s}_{\textsf{ref}}}\rangle },{\langle {i},{{i}_{\textsf{ref}}}\rangle }\rangle \models {\textsf{init}}_{\textsf{ref}}$, and
3. for all $s: X\rightarrow \textsf{Val}$ and ${s}_{\textsf{ref}}': {X}_{\textsf{ref}}'\rightarrow \textsf{Val}$ such that $\langle {s},{{s}_{\textsf{ref}}}\rangle \models {\textsf{final}}_{\textsf{ref}}$ we have $s \models \textsf{final}$, and for every $s: X\rightarrow \textsf{Val}$ such that $s \models \textsf{final}$ and every ${s}_{\textsf{ref}}: {X}_{\textsf{ref}}\rightarrow \textsf{Val}$ we have $\langle {s},{{s}_{\textsf{ref}}}\rangle \models {\textsf{final}}_{\textsf{ref}}$.

**Fig. 4:**
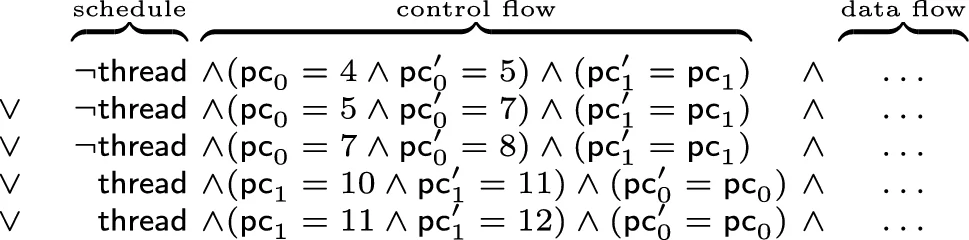
Deterministic transition relation for Listing 1.1

**Fig. 5:**
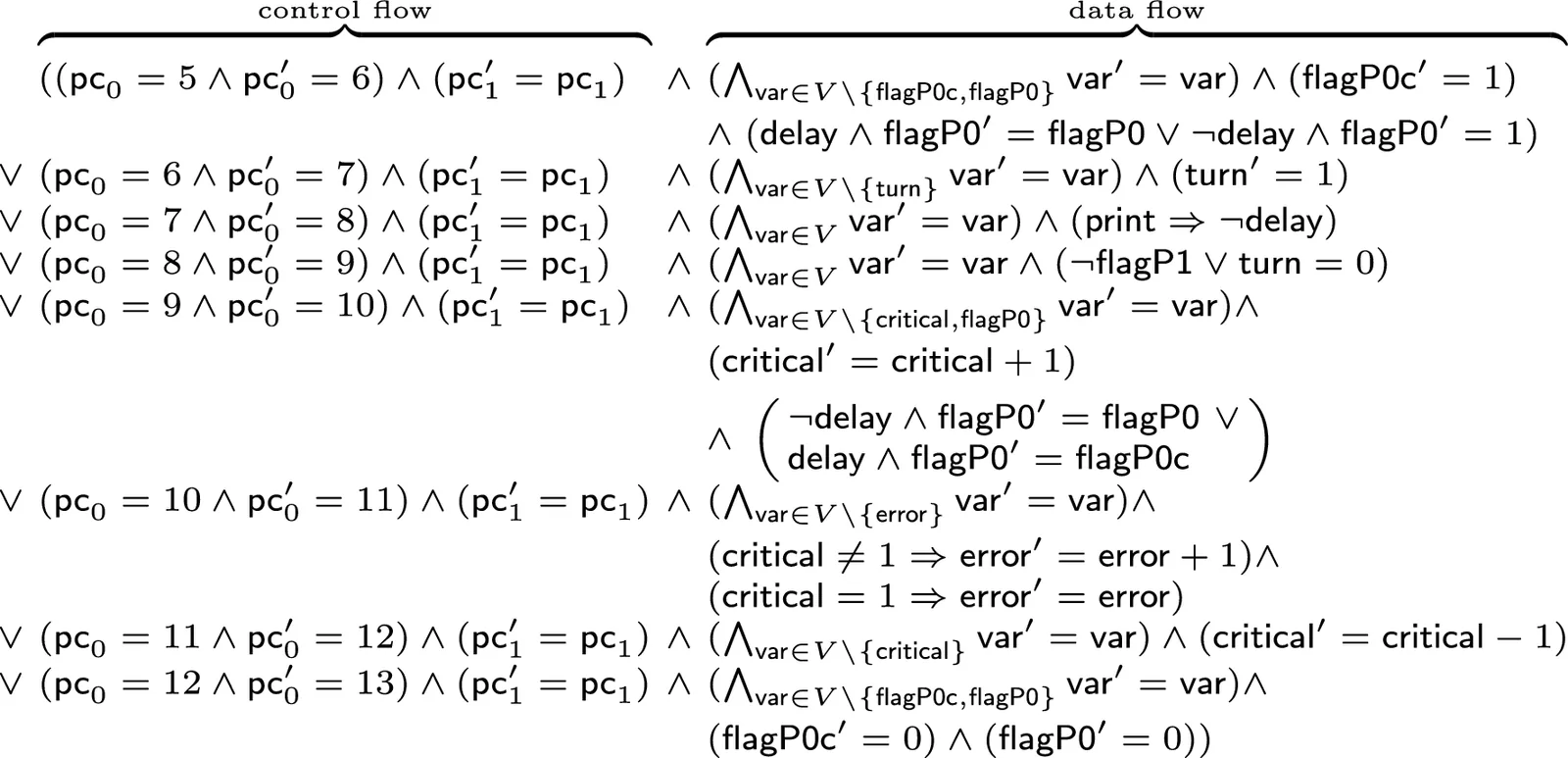
A part of ${T}_{\textsf{ref}}$ for Listing 1.3 (where $V{\mathop {=}\limits ^{\tiny def }}(X{\uplus }{X}_{\textsf{ref}})\setminus \{\textsf{pc}_0,\textsf{pc}_1\}$)

We note that the above definition preserves executions: Let ${S}_{\textsf{ref}}$ be a refinement of some STS *S*. Then every execution of ${S}_{\textsf{ref}}$ gives rise to an execution of *S* by projecting away the additional state and input variables. On the other hand, the conditions in the refinement definition ensure that every execution of *S* can be extended to an execution of ${S}_{\textsf{ref}}$ by choosing suitable values for the additional state and input variables. We note that refinements can be thought of as adding additional information to the STS under analysis, and the requirements in our definition ensure that executions are preserved. If *all* mechanisms (i.e., sources of nondeterminism) are explicit, refinement yields a deterministic system:

#### Definition 9

(Determinization) Given some STS $S=(X, I, \textsf{init}, \textsf{final}, T)$, we call an STS ${S}_{\textsf{det}} {\mathop {=}\limits ^{\tiny def }}(X \uplus {X}_{\textsf{det}}, I \uplus M, {\textsf{init}}_{\textsf{det}}, {\textsf{final}}_{\textsf{det}}, {T}_{\textsf{det}})$ a *determinization* of *S*, if ${S}_{\textsf{det}}$ is a refinement of *S* and ${S}_{\textsf{det}}$ is input-deterministic. We further call *M* the *mechanisms* of the determinization.

#### Example 8

The Therac-25 transition system from Example 4 can be refined by setting ${I}_{\textsf{ref}} = \{\textsf{thread}\}$, where $\textsf{thread}$ is a Boolean variable selecting which thread takes a step. The refined transition relation is shown in Fig. 4.

#### Example 9

The floating point transition system from Example 5 can be extended to a deterministic system by setting ${I}_{\textsf{ref}} = \{\textsf{debug}\}$ and considering the refined initial condition $(\textsf{pc} = 3 \wedge \textsf{v} = 10^{308} \wedge \textsf{y} = 0 \wedge (\textsf{debug} \Leftrightarrow \textsf{print}))$, and leaving the transition relation unchanged. We point out that the initial value of the input variable $\textsf{debug}$ fixes the value of $\textsf{print}$, which in turn fixes the transition relation reflecting the presence of the printf-statement.

For Example 8 and Example 9, we have ${X}_{\textsf{ref}}=\emptyset$. In the Peterson example below, the refinement contains a state variable reflecting whether the cache state has been propagated to main memory.

#### Example 10

Peterson’s algorithm (Listing 1.3) can be modeled as a determinisitic STS. In this example we present the final refinement that makes all involved mechanisms explicit. Alternatively, the mechanisms could be made explicit in successive refinement steps. Figure 5 shows the part of the transition relation that models $\textsf{P0}$.

Let $X = \{\textsf{pc}_0,\textsf{pc}_1,\textsf{flagP0}, \textsf{flagP0c},[1] \textsf{flagP1}, \textsf{flagP1c},[1] \textsf{turn}, \textsf{critical}, \textsf{error}, \textsf{print}\}$ where $\textsf{flagP0c}$ and $\textsf{flagP1c}$ represent the locally cached versions of the flags. We have *I* = ∅ and ${I}_{\textsf{ref}} = \{\textsf{thread},\textsf{debug},\textsf{reorder}\}$, where $\textsf{thread}$ indicates whether $\textsf{P0}$ or $\textsf{P1}$ takes a step ($\textsf{thread}$ is omitted in Fig. 5). Let ${X}_{\textsf{ref}}=\{\textsf{delay}\}$, and let ${\textsf{init}}_{\textsf{ref}}$ imply $(\textsf{print}=\textsf{debug}\wedge \textsf{delay}=\textsf{reorder})$. The variable $\textsf{print}$ indicates that the program version with $\texttt {printf}$-debugging is executed, and $\textsf{delay}$ is true if the modifications of the flags $\textsf{flagP0}$ and $\textsf{flagP1}$ are only committed to shared memory after entering the critical section (to avoid clutter, we assume only two possible points for committing the modification of $\textsf{flagP0}$). We use $(\textsf{print}\Rightarrow \lnot \textsf{delay})$ to model the interplay between two mechanisms where the printf instruction prevents reordering because of the added barrier, resulting in a partial transition relation. Moreover, ${\textsf{init}}_{\textsf{ref}}$ ensures that $\textsf{flagP0} = \textsf{flagP0c} = \textsf{flagP1} = \textsf{flagP1c} = \textsf{turn} = \textsf{critical} = \textsf{error} = 0$ and $\textsf{pc}_0=5$ and $\textsf{pc}_1=15$, and ${\textsf{final}}_{\textsf{ref}}$ is $\textsf{pc}_0 = 13 \wedge \mathsf {pc_1} = 23$.

Note that the processor running the original nondeterministic version of Peterson’s algorithm already has micro-architectural features that facilitate instruction reordering (not modeled in Example 10); the auxiliary input $\textsf{reorder}$ and variable $\textsf{delay}$ merely make this mechanism observable.

### Defining causes

In the following, we provide a formal definition of causes inspired by Lewis’ counterfactuals [36] and the causality framework of Galles and Pearl [22].

#### Definition 10

(Cause) Let $S=(X, I, \textsf{init}, \textsf{final}, T)$ be a nondeterministic STS with a Heisenbug (Definition 7) with respect to an assertion φ, and let ${S}_{\textsf{det}} {\mathop {=}\limits ^{\tiny def }}(X \uplus {X}_{\textsf{det}}, I \uplus M, {\textsf{init}}_{\textsf{det}}, {\textsf{final}}_{\textsf{det}}, {T}_{\textsf{det}})$ be a determinization of *S*. Let $M=M_C\uplus M_N$. We say that $M_C$ is a *cause* with respect to *M* and φ iff1and $M_C$ is a minimal subset of *M* with this property.

We note that in the above definition we require the inputs *I* to agree on the executions $\pi _c$ and $\pi _w$, while only the inputs *M* may differ. The rationale is that we want to apply this definition for studying the causes of Heisenbugs: We are given some (nondeterministic) STS *S* with inputs *I*, which has a Heisenbug. We now consider some determinization ${S}_{\textsf{det}}$ of *S* to which we have added inputs *M*, modeling the *mechanisms* responsible for the nondeterminism. The above definition then allows to study the cause among the modeled mechanisms: A subset $M_C \subseteq M$ is a cause of a Heisenbug, if the Heisenbug still occurs when the inputs $M_N$ agree in the deviating executions $\pi _c$ and $\pi _w$.

#### Proposition 1

(Existence of Nonempty Cause) Given a nondeterministic STS $S=(X, I, \textsf{init}, \textsf{final}, T)$ with a Heisenbug (Definition 7) with respect to an assertion φ, let ${S}_{\textsf{det}} {\mathop {=}\limits ^{\tiny def }}(X \uplus {X}_{\textsf{det}}, I \uplus M, {\textsf{init}}_{\textsf{det}}, {\textsf{final}}_{\textsf{det}}, {T}_{\textsf{det}})$ be a determinization of *S*. Then there exists a cause $M_C \subseteq M$ w.r.t. φ. Moreover, all causes $M_C$ are nonempty.

#### Proof

Let $\pi _c$ and $\pi _w$ be executions of *S* that satisfy Definition 7, i.e., ${I}({\pi _c}) = {I}({\pi _w})$, $\pi _c \not \models \varphi$, and $\pi _w \models \varphi$. Since refinements preserve executions, there must be executions ${\pi _c}_{\textsf{det}}$ and ${\pi _w}_{\textsf{det}}$ of ${S}_{\textsf{det}}$ such that ${(I{\uplus } X)}({{\pi _c}_{\textsf{det}}})=\pi _c$ and ${(I{\uplus } X)}({{\pi _w}_{\textsf{det}}})=\pi _w$. In particular, ${I}({{\pi _c}_{\textsf{det}}}) = {I}({{\pi _w}_{\textsf{det}}})$, ${\pi _c}_{\textsf{det}} \not \models \varphi$ and ${\pi _w}_{\textsf{det}} \models \varphi$. Let $M_N \subseteq M$ such that $m \in M_N$ if and only if ${m}({{\pi _c}_{\textsf{det}}}) = {m}({{\pi _w}_{\textsf{det}}})$ and $M_C = M \setminus M_N$. Assume that $\pi _c$, ${\pi _c}_{\textsf{det}}$, $\pi _w$, and ${\pi _w}_{\textsf{det}}$ are chosen such that $M_C$ is minimal. Then $M_C$ is a cause with respect to *M* and φ.

Now assume that there exists a cause $M_C = \emptyset$. Therefore, $M = M_N$ and ${(I \uplus M)}({{\pi _c}_{\textsf{det}}}) = {(I \uplus M)}({{\pi _w}_{\textsf{det}}})$. Let $\langle s_c,{s_c}_{\textsf{det}}\rangle$ and $\langle s_w,{s_w}_{\textsf{det}}\rangle$ be the initial states of ${\pi _c}_{\textsf{det}}$ and ${\pi _w}_{\textsf{det}}$, respectively. Since ${S}_{\textsf{det}}$ is input-deterministic, there is at most one state with $\langle \langle s,{s}_{\textsf{det}}\rangle ,\langle i,m\rangle \rangle \models {\textsf{init}}_{\textsf{det}}$, hence $\langle s_c,{s_c}_{\textsf{det}}\rangle =\langle s_w,{s_w}_{\textsf{det}}\rangle$. Moreover, for every state $\langle s,{s}_{\textsf{det}}\rangle$, each input $\langle i,m\rangle$ determines a unique successor state $\langle s',{s}_{\textsf{det}}'\rangle$. By induction, it follows that ${\pi _c}_{\textsf{det}}={\pi _w}_{\textsf{det}}$. This yields a contradiction to the fact that ${\pi _c}_{\textsf{det}} \not \models \varphi$ and ${\pi _w}_{\textsf{det}} \models \varphi$. Hence $M_C$ must be nonempty. □

#### Example 11

The Peterson example contains a Heisenbug with respect to $\varphi {\mathop {=}\limits ^{\tiny def }}(\textsf{error} = 0)$. An analysis of the subsets of the set of mechanisms $\{\textsf{thread},\textsf{debug},\textsf{reorder}\}$ (cf. Example 10) detects that $\{\textsf{reorder}\}$ and $\{\textsf{thread}\}$ are causes, but $\{\textsf{debug}\}$ is not: The set $\{\textsf{reorder}\}$ is a cause because of two executions which both have $\textsf{debug} = \textsf{false}$ and the same schedule interleaving the critical sections, but only one execution sets $\textsf{reorder} = \textsf{true}$ and hence exhibits the bug. The set $\{\textsf{thread}\}$ is a cause because of two executions which both have $\textsf{debug} = \textsf{false}$ and $\textsf{reorder} = \textsf{true}$ where one execution uses a sequential schedule of the two processes and the second execution uses a schedule interleaving the critical sections. Only the second execution exhibits the bug. However, the set $\{\textsf{debug}\}$ is not a cause because any two executions would either both have to set $\textsf{reorder} = \textsf{false}$, making the bug impossible or both set $\textsf{reorder} = \textsf{true}$. In this case, by contraposition the constraint $(\textsf{print}\Rightarrow \lnot \textsf{delay})$ enforces $\textsf{debug} = \textsf{false}$ (since $\textsf{debug}=\textsf{print}$ and $\textsf{delay}=\textsf{reorder}$ thanks to ${\textsf{init}}_{\textsf{ref}}$), yielding a bug in both executions if the schedule interleaves the critical sections or in no execution otherwise.

### Causes and nondeterminism

By introducing the notion of a *contributing cause* below, we show that even in the presence of nondeterminism we can still provide guarantees.

#### Definition 11

(Contributing Cause) Let $S=(X, I, \textsf{init}, \textsf{final}, T)$ be a nondeterministic STS with a Heisenbug (Definition 7) with respect to an assertion φ, and let ${S}_{\textsf{ref}} = (X \uplus {X}_{\textsf{ref}}, I \uplus M, {\textsf{init}}_{\textsf{ref}}, {\textsf{final}}_{\textsf{ref}}, {T}_{\textsf{ref}})$ be a (potentially nondeterministic) refinement of *S*. We now choose some partitioning $M = M_C \uplus M_N$ that satisfies Equation 1 in Definition 10, such that $M_C$ is a minimal set with this property. We call $M_C$ a *contributing cause* of a Heisenbug.

We argue that any contributing cause must be a subset of a nonempty cause in a corresponding determinization:

#### Theorem 1

Let $S=(X, I, \textsf{init}, \textsf{final}, T)$ be a nondeterministic STS with a Heisenbug (Definition 7) with respect to an assertion φ, let ${S}_{\textsf{ref}} = (X \uplus {X}_{\textsf{ref}}, I \uplus M, {\textsf{init}}_{\textsf{ref}}, {\textsf{final}}_{\textsf{ref}}, {T}_{\textsf{ref}})$ be a (potentially nondeterministic) refinement of *S*, and let ${S}_{\textsf{det}} = (X \uplus {X}_{\textsf{ref}} \uplus {X}_{\textsf{det}}, I \uplus M \uplus J, {\textsf{init}}_{\textsf{det}}, {\textsf{final}}_{\textsf{det}}, {T}_{\textsf{det}})$ be a determinization of ${S}_{\textsf{ref}}$. Let $M_C \subseteq M$ be a contributing cause in ${S}_{\textsf{ref}}$ with respect to assertion φ. Then, there exists a nonempty cause $C \subseteq M \uplus J$ in ${S}_{\textsf{det}}$ such that $M_C \subseteq C \setminus J$.

#### Proof

Let $\pi _c$ and $\pi _w$ be executions of *S* that satisfy Definition 7, i.e., ${I}({\pi _c}) = {I}({\pi _w})$, $\pi _c \not \models \varphi$, and $\pi _w \models \varphi$. Since refinements preserve executions, there must be executions ${\pi _c}_{\textsf{ref}}$ and ${\pi _w}_{\textsf{ref}}$ of ${S}_{\textsf{ref}}$, and executions ${\pi _c}_{\textsf{det}}$ and ${\pi _w}_{\textsf{det}}$ of ${S}_{\textsf{det}}$ such that ${(I \uplus X)}({{\pi _c}_{\textsf{ref}}})=\pi _c$ and ${(I \uplus X)}({{\pi _w}_{\textsf{ref}}})=\pi _w$ as well as ${(I \uplus M \uplus X \uplus {X}_{\textsf{ref}} )}({{\pi _c}_{\textsf{det}}})={\pi _c}_{\textsf{ref}}$ and ${(I \uplus M \uplus X \uplus {X}_{\textsf{ref}})}({{\pi _w}_{\textsf{det}}})={\pi _w}_{\textsf{ref}}$. By Definition 11, for $M_N = M \setminus M_C$ it holds that ${(I{\uplus } M_N)}({{{\pi _c}_{\textsf{ref}}}})={(I{\uplus } M_N)}({{{\pi _w}_{\textsf{ref}}}})$. We note that the above implies that also ${(I{\uplus } M_N)}({{\pi _c}_{\textsf{det}}})={(I{\uplus } M_N)}({{\pi _w}_{\textsf{det}}})$. Following an argument similar to the one for Proposition 1, there must be a nonempty subset $C \subseteq (M_C \uplus J)$ such that ${C}({{\pi _c}_{\textsf{det}}})\not ={C}({{\pi _w}_{\textsf{det}}})$, i.e., there exists a nonempty cause *C* satisfying Definition 10 with $C\subseteq M_C {\uplus } J$.

Now assume that $M_C \not \subseteq C$. Then $M_C$ is not minimal, since $(M_C \cap C)$ also constitutes a contributing cause. Thus, we must have $M_C \subseteq C \setminus J$. □

#### Example 12

The refined STS in Example 8 is nondeterministic as the initial values of $\textsf{filter}$ and $\textsf{highEnergy}$ are unconstrained. Following Definition 11, $\{\textsf{thread}\}$ is a contributing cause. Consider a further refinement with ${I}_{\textsf{ref}} = \{\textsf{initF}, \textsf{initH}\}$ and $\textsf{init} =(\textsf{filter} = \textsf{initF} \wedge \textsf{highEnergy} = \textsf{initH} \wedge \textsf{isXray}\wedge \textsf{isHigh} \wedge \textsf{pc}_0 = 4 \wedge \textsf{pc}_1 = 10)$. As the initial values are never read, the cause is again $\{\textsf{thread}\}$.

We provide a condition under which contributing causes are also causes:

#### Definition 12

(Cause in the Presence of Nondeterminism) Let $S=(X, I, \textsf{init}, \textsf{final}, T)$ be a nondeterministic STS with a Heisenbug (Definition 7) with respect to an assertion φ, and let ${S}_{\textsf{ref}} = (X \uplus {X}_{\textsf{ref}}, I \uplus M, {\textsf{init}}_{\textsf{ref}}, {\textsf{final}}_{\textsf{ref}}, {T}_{\textsf{ref}})$ be a (potentially nondeterministic) refinement of *S*, such that for all traces $\pi ,\pi '$ of ${S}_{\textsf{ref}}$ with ${I{\uplus } M}({\pi })={I{\uplus } M}({\pi '})$ we have that

1. π ends in a final state if and only if $\pi^{\prime }$ ends in a final state,
2. $\pi \models \varphi$ if and only if $\pi ' \models \varphi$.

Let φ, $M = M_C \uplus M_N$ satisfy Equation 1 in Definition 10, and $M_C$ is a minimal set with this property. Then, we say that $M_C$ is a *cause in presence of nondeterminism* with respect to *M* and φ.

We will next state a justification for the introduction of the above definition. We first establish that input-enabled determinizations always exist:

#### Proposition 2

Let $S=(X, I, \textsf{init}, \textsf{final}, T)$ be an STS. Then, an input-enabled deterministic refinement ${S}_{\textsf{ref}} = (X \uplus {X}_{\textsf{ref}}, I \uplus {I}_{\textsf{ref}}, {\textsf{init}}_{\textsf{ref}}, {\textsf{final}}_{\textsf{ref}}, {T}_{\textsf{ref}})$ of *S* always exists.

#### Proof

We set $${I}_{\textsf{ref}} = \{\textsf{oracle}\}$$ for a single variable $$\textsf{oracle}$$, whose values are mappings of configurations to successors, i.e., $$\textsf{oracle}$$ fixes a successor state $s^{\prime }$ for every configuration $$\langle {s},{i}\rangle $$ such that $$\langle {\langle }{s},{i}{\rangle },{s'}\rangle \models T$$). We then adopt $${T}_{\textsf{ref}}$$ from *T* as the transition relation that moves to the successor state fixed by the oracle variable. Likewise, we adopt the initial condition $${\textsf{init}}_{\textsf{ref}}$$. We note that this construction does not require any additional variables and we have $${X}_{\textsf{ref}} = \emptyset $$ and $${\textsf{final}}_{\textsf{ref}} = \textsf{final} $$. Clearly, the above construction ensures that $${S}_{\textsf{ref}}$$ is input-deterministic. However, $${S}_{\textsf{ref}}$$ does not need to be input-enabled. We can additionally ensure that $${S}_{\textsf{ref}}$$ is input-enabled by adding a boolean variable, i.e., setting $${X}_{\textsf{ref}} = \{\textsf{z}\}$$, for some fresh boolean-valued variable $$\textsf{z}$$, which denotes whether the computation has gotten stuck. That is, we set $$\textsf{z}$$ to false in $${\textsf{init}}_{\textsf{ref}}$$. We modify the transition relation $${T}_{\textsf{ref}}$$ to set $$\textsf{z}$$ to true, in case *T* does not have a successor from the current state (the remaining variables can be set arbitrarily); for all states where $$\textsf{z}$$ is true, the transition relation $${T}_{\textsf{ref}}$$ is defined to stay in this state for every input (cf. [18, Section 3.1]). The above ensures that the transition relation $${T}_{\textsf{ref}}$$ is total. Finally, we modify $${\textsf{final}}_{\textsf{ref}}$$ to require that $$\textsf{z}$$ is false, which ensures that stuck traces are not considered as executions. □

We next establish that no matter the input-enabled determinization $S^{\prime }$ of an STS *S*, a cause in the presence of nondeterminism in *S* is always a cause in $S^{\prime }$. Together with Proposition 2, which guarantees the existence of an input-enabled determinization (at least in theory), we obtain that a cause in presence of nondeterminism can indeed be considered as a cause.

#### Theorem 2

Let $S=(X, I, \textsf{init}, \textsf{final}, T)$ be a nondeterministic STS with a Heisenbug (Definition 7) with respect to an assertion φ, let ${S}_{\textsf{ref}} = (X \uplus {X}_{\textsf{ref}}, I \uplus M, {\textsf{init}}_{\textsf{ref}}, {\textsf{final}}_{\textsf{ref}}, {T}_{\textsf{ref}})$ be a (potentially nondeterministic) refinement of *S*, and let $M_C \subseteq M$ be a cause in presence of nondeterminism. Further, let ${S}_{\textsf{det}} {\mathop {=}\limits ^{\tiny def }}(X \uplus {X}_{\textsf{ref}} \uplus {X}_{\textsf{det}}, I \uplus M \uplus J, {\textsf{init}}_{\textsf{det}}, {\textsf{final}}_{\textsf{det}}, {T}_{\textsf{det}})$ be an input-enabled determinization of ${S}_{\textsf{ref}}$. Then $M_C$ is a nonempty cause in ${S}_{\textsf{det}}$.

#### Proof

Let $$\pi _c$$ and $$\pi _w$$ be executions of *S* that satisfy Definition 7, i.e., $${I}({\pi _c}) = {I}({\pi _w})$$, $$\pi _c \not \models \varphi $$, and $$\pi _w \models \varphi $$. Since refinements preserve executions, there must be executions $${\pi _c}_{\textsf{ref}}$$ and $${\pi _w}_{\textsf{ref}}$$ of $${S}_{\textsf{ref}}$$ such that $${(I \uplus X)}({{\pi _c}_{\textsf{ref}}})=\pi _c$$ and $${(I \uplus X)}({{\pi _w}_{\textsf{ref}}})=\pi _w$$. Because $${S}_{\textsf{det}}$$ refines $${S}_{\textsf{ref}}$$ there must be an execution $${\pi _c}_{\textsf{det}}$$ such that $${(I \uplus M \uplus X \uplus {X}_{\textsf{ref}} )}({{\pi _c}_{\textsf{det}}})={\pi _c}_{\textsf{ref}}$$. In particular, we have $${\pi _c}_{\textsf{det}}\not \models \varphi $$. Because $${S}_{\textsf{det}}$$ is input-enabled, we can obtain a trace π of $${S}_{\textsf{det}}$$ such that $${J}({\pi })={J}({{\pi _c}_{\textsf{det}}})$$ and $${(I \uplus M \uplus X \uplus {X}_{\textsf{ref}})}({{\pi }_{\textsf{det}}})={\pi _w}_{\textsf{ref}}$$. Note that π induces a trace $\pi^{\prime }$ of $${S}_{\textsf{ref}}$$ with $$\pi '={(I\uplus M \uplus X \uplus {X}_{\textsf{ref}})}({{\pi _w}_{\textsf{det}}})$$. Hence, by the assumptions stated in Definition 12, the trace $\pi^{\prime }$ is in fact an execution (i.e., ends with a final configuration), and we have $$\pi ' \models \varphi $$. Thus, we also get that π is an execution and that we have $$\pi \models \varphi $$. Thus, we have shown that the pair $${\pi _c}_{\textsf{det}}$$ and π witnesses that $$M_C$$ satisfies Equation 1 of Definition 10 in $${S}_{\textsf{det}}$$. Note that this holds for any $$M_C$$ induced by some traces $$\pi _c$$ and $$\pi _w$$ that witness a Heisenbug. In particular, this holds for the $$M_C$$ that is minimal w.r.t. the definition of a cause in presence of nondeterminism. We now fix such a minimal $$M_C$$ and claim that it is indeed a cause in $${S}_{\textsf{det}}$$. Assume otherwise. Then there is some $$N \subsetneq M_C$$ such that *N* is a cause in $${S}_{\textsf{det}}$$. In particular, there are some executions $${\pi _c}_{\textsf{det}}$$ and $${\pi _w}_{\textsf{det}}$$ of $${S}_{\textsf{det}}$$ with $${\pi _c}_{\textsf{det}} \not \models \varphi $$ and $${\pi _w}_{\textsf{det}} \models \varphi $$, and $$(I \uplus M \setminus N \uplus J)({\pi _c}_{\textsf{det}}) = (I \uplus M \setminus N \uplus J)({\pi _w}_{\textsf{det}})$$. However, as $${S}_{\textsf{det}}$$ refines $${S}_{\textsf{ref}}$$, we then get that $${(I \uplus M \uplus X \uplus {X}_{\textsf{ref}})}({{\pi _c}_{\textsf{det}}})$$ and $${(I \uplus M \uplus X \uplus {X}_{\textsf{ref}})}({{\pi _w}_{\textsf{det}}})$$ are two executions that witness that *N* is a cause in the presence of nondeterminism. This violates the minimality of $$M_C$$. Hence, $$M_C$$ is a cause in $${S}_{\textsf{det}}$$. We note that this also implies that $$M_C$$ is nonempty by Proposition 1. □

#### Example 13

The nondeterministic refinement of the Therac-25 STS in Example 8 satisfies the properties in Definition 12. The refinement in Example 12 is input-enabled and deterministic, and the contributing cause is indeed a cause.

### Testing and causal analysis

In the context of testing, an evaluation of Definition 10 and Definition 11, respectively, is limited to the subset of the executions induced by a given test suite. Proposition 1 characterizes the conclusions that can be drawn by analyzing a subset of the executions of an STS:

#### Lemma 1

Let $\pi _c$ and $\pi _w$ be executions satisfying Equation 1 in Definition 10 (or Definition 11, respectively) and let $M_C$ be the inputs deviating in $\pi _c$ and $\pi _w$. Then $M_C$ is a superset of a cause (or contributing cause, respectively).

#### Proof

Note that $M_C$ is a cause according to Definition 10 (or a contributing cause according to Definition 11) if it is minimal with respect to Equation 1. Hence, either $M_C$ is minimal w.r.t. Equation 1 and thus a cause, or there is a cause $N \subsetneq M_C$. □

Proposition 1 provides guarantees even if an exhaustive analysis is infeasible. If, in addition, the conditions in Definition 12 are met (i.e., we can control or at least observe the relevant mechanisms), then Proposition 2, Theorem 2, and Proposition 1 guarantee that each overapproximation of a cause identified by testing includes a *nonempty* (contributing) cause.

### Correlated causes

In previous examples (such as Example 11), causes typically contain a single mechanism. In general, this need not be the case: in Definition 10, the transition relation can induce a correlation between two mechanism by either

1. ruling out certain combinations of values of nondeterministic variables by means of partial transitions (as in Example 14 below), or
2. introducing nonterminating traces that fail to reach the relevant assertion φ for certain combinations of values of nondeterministic variables. This is illustrated in the Example 15 below.

Example 14 illustrates that a cause derived from Definition 10 or Definition 12 may be a set of mechanisms if the transition relation is partial:

#### Example 14

Consider a deterministic STS with $I = \{\textsf{c}, \textsf{d}\}$ (where $0\le \textsf{c},\textsf{d} \le 2$), $X = \{\textsf{x}, \textsf{y}, \textsf{pc}\}$, initial condition $(\textsf{pc} = 0\wedge \textsf{x} = 0\wedge \textsf{y}=0)$, final condition $(\textsf{pc} = 1)$ and transition relation $(\textsf{pc} = 0\wedge (\textsf{c}=2\Leftrightarrow \textsf{d}=2) \wedge \textsf{x}' = \textsf{c} \wedge \textsf{y}' = \textsf{d} \wedge \textsf{pc}' = 1)$. Let $\varphi \equiv (\textsf{x}<2)\vee (\textsf{y}<2)$. The set $\{\textsf{c}\}$ is not a cause, as fixing the value of $\textsf{d}$ for both executions automatically also fixes the outcome. Similarly, $\{\textsf{d}\}$ is not a cause. However, the set $\{\textsf{c}, \textsf{d}\}$ is a cause.

Example 15, on the other hand, demonstrates that a dependency between inputs can also be introduced by nonterminating traces (excluded in the premise of Definition 12):

**Figure:**
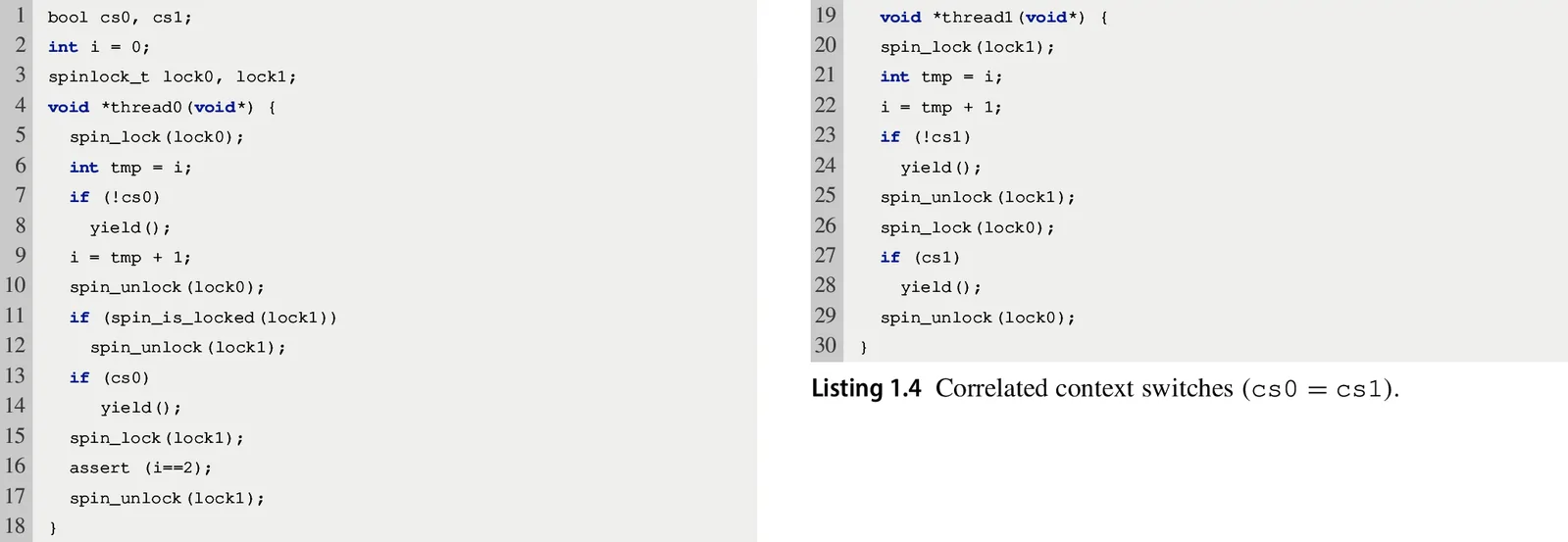

#### Example 15

Consider the code snippet in Listing 1.4. The variables $\texttt {cs0}$ and $\texttt {cs1}$ control the context switches in lines 8, 14, 24, and 28. The function spin_lock is implemented using busy wait. Consider the executions where $\texttt {cs0}=\texttt {cs1}=0$ and $\texttt {cs0}=\texttt {cs1}=1$ (assume that thread0 executes first). The first execution triggers a race condition on i, making the assertion in line 16 fail. The second execution, on the other hand, does not violate the assertion. For $\texttt {cs0}$ or $\texttt {cs1}$ to be causes independently, $\texttt {cs0}\ne \texttt {cs1}$ must hold. Consider $\texttt {cs0}=0$ and $\texttt {cs1}=1$ first. Then the context switch in line 28 (and hence the assertion in line 16) is never reached, since thread 0 still holds lock0 when thread 1 reaches line 26. Alternatively, consider $\texttt {cs0}=1$ and $\texttt {cs1}=0$. Then lock1 is still held by thread1 after the context switch in line 24, causing thread0 to block before the assertion in 16 is reached. Consequently, neither of these two (nonterminating) traces satisfies Definition 10.

### Singleton cause sets

While Sect. 3.5 demonstrates that causes can be sets of mechanisms in general, the causes $\{\textsf{reorder}\}$ and $\{\textsf{thread}\}$ in Example 11 are singleton sets. This raises the question whether there are conditions under which causes are always singleton. The following theorem states that causes are always singleton sets in case there is an input-enabled determinization such that *all traces reach a final state after the same number of steps*:

#### Theorem 3

Let $S=(X, I, \textsf{init}, \textsf{final}, T)$ be a nondeterministic STS with a Heisenbug (Definition 7) with respect to an assertion φ, and let ${S}_{\textsf{det}} {\mathop {=}\limits ^{\tiny def }}(X \uplus {X}_{\textsf{det}}, I \uplus M, {\textsf{init}}_{\textsf{det}}, {\textsf{final}}_{\textsf{det}}, {T}_{\textsf{det}})$ be an input-enabled determinization of *S* such that all traces reach a final state after the same number of steps. Then all cause sets $M_C \subseteq M$ are singleton.

#### Proof

Because *S* has a Heisenbug, there is some nonempty cause $M_C$ by Proposition 1 and two executions $\pi _w$ and $\pi _c$ shaped as described in Definition 10. In particular, $\pi _c\not \models \varphi$ and $\pi _w \models \varphi$. Now assume that $M_C$ has cardinality ≥2. We arbitrarily partition $M_C = \{\textsf{c}\} \uplus D$ for some mechanism $\textsf{c}$ and set of mechanisms *D*. We then construct an execution π by setting 1) ${M_N}({\pi }){\mathop {=}\limits ^{\tiny def }}{M_N}({\pi _c}) = {M_N}({\pi _w})$, 2) ${I}({\pi }){\mathop {=}\limits ^{\tiny def }}{I}({\pi _c}) = {I}({\pi _w})$, 3) ${\textsf{c}}({\pi }){\mathop {=}\limits ^{\tiny def }}{\textsf{c}}({\pi _c})$ and 4) ${D}({\pi }){\mathop {=}\limits ^{\tiny def }}{D}({\pi _w})$. We note that because ${S}_{\textsf{det}}$ is input-enabled, we can indeed construct such a trace. Further, we have by assumption that π reaches a final state after the same number of steps as $\pi _c$ and $\pi _w$. As by our assumption $M_C$ is a cause set of minimal cardinality, the set $\{\textsf{c}\}$ cannot be a cause (as it is of smaller cardinality). Hence, $\pi \not \models \varphi$ (because of $\pi _c\not \models \varphi$ as well as 1), 2) and 3)). As by our assumption $M_C$ is a cause set of minimal cardinality, the set *D* cannot be a cause (as it is of smaller cardinality). Hence, $\pi \models \varphi$ (because of $\pi _w \models \varphi$ as well as 1), 2) and 4)). This yields a contradiction. Hence, every cause set $M_C$ must have cardinality one. □

These conditions may have practical implications on how to debug Heisenbugs, as they allow causality to be decided by checking every element in the set of nondeterministic mechanisms individually (instead of each subset). Note that the conditions are sufficient but not necessary: all causes in Example 11 are singleton sets, even though the transition relation in Fig. 5 is partial. Example 14 illustrates why the theorem does not hold for systems which are not input-enabled (i.e., where inputs are constrained). However, Theorem 3 demonstrates that it is possible to define sufficient conditions that avoid that mechanisms become “entangled” in the sense of Sect. 3.5. We leave the investigation of further settings when causes are guaranteed to be singleton for future work.

## Heisenbugs and quiescence

The system model in Definition 1 allows quiescent inputs with a special value τ in order to model the timing in which inputs are provided by the user and processed by the system. Both the writing of the inputs and the reading might be delayed due to various reasons not observable or controllable by the user. In this chapter, we study Heisenbugs that arise from this gap in observability.

### Example 16

The program in Listing 1.5 concurrently executes two procedures,  for processing inputs and  for actually storing the data. Assume the  models a nonblocking read: the function returns the value of the current input if it is nonquiescent (i.e., not τ), and otherwise the value passed as its argument.

**Figure:**
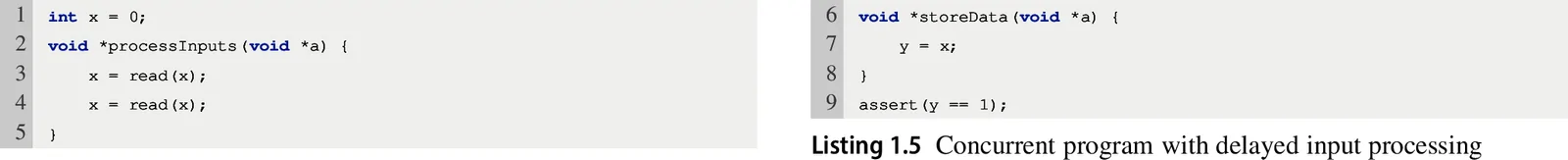

**Fig. 6:**
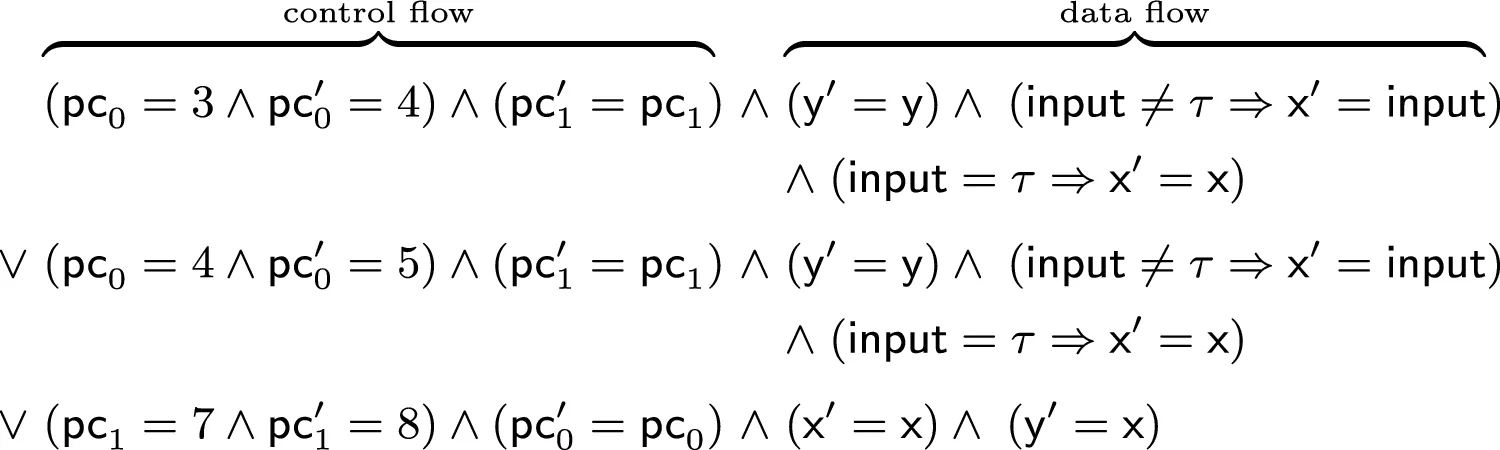
Transition relation for Listing 1.5

We can formally describe the program as an STS with $I = \{\textsf{input}\}$, $X = \{\textsf{x}, \textsf{y}, \mathsf {pc_0}, \mathsf {pc_1}\}$ where $\mathsf {pc_0}$ and $\mathsf {pc_1}$ model the program counters of the two threads, initial condition $(\textsf{x} = 0 \wedge \mathsf {pc_0} = 3 \wedge \mathsf {pc_1} = 7)$, final condition $(\mathsf {pc_0} = 5 \wedge \mathsf {pc_1} = 8)$ and transition relation as shown in Fig. 6.

The final value of the variable $\textsf{y}$ depends on the input for $\textsf{x}$, but also on the timing in which the input is provided and the schedule that defines in which order the instructions are executed. We fix a schedule where  completes before  is run, i.e., the reads in lines 3 and 4 execute before the assignment in line 7. Now consider the execution $\pi _w$ in which $\textsf{input}(\pi _w) = 2, 1, \tau$, i.e., the user provides inputs 1 and 2 in the first two steps of the execution. In this case the assertion in line 9 holds. Now consider another execution $\pi _c$ with the same schedule but $\textsf{input}(\pi _c) = 2, \tau , 1$ that might for example arise if the user takes some time to provide the input value 1. This will lead the assertion to fail as the value is only provided after the completion of .

Note, however, that the two executions in the example are not counterexample and witness executions of a Heisenbug according to Definition 7 as the two executions differ in the order of quiescent and nonquiescent values. This might not be observable by the user as captured in the following definition.

### Definition 13

(Observable Input Sequence) Let π be a trace of the STS $(X, I, \textsf{init}, \textsf{final}, T)$, and let i∈I be some input variable. The *observable input sequence* $\textsf{obs}_{i}({\pi })$ is defined inductively:

$$\begin{aligned} \textsf{obs}_{i}({\varepsilon }) = \varepsilon , \qquad \textsf{obs}_{i}({c \cdot \pi }) =\left\{ \begin{array}{ll} \textsf{obs}_{i}({\pi }), & \text {if~} {c.i} = \tau \\ {c.i} \cdot \textsf{obs}_{i}({\pi }) &  \text {otherwise}\\ \end{array}\right. \end{aligned}$$

where ε is the trace of length zero, *c*.*i* represents the value of input *i* in configuration *c*, and · represents concatenation.

### Example 17

For the executions introduced in Example 16 we have $\textsf{obs}_{\textsf{input}}({\pi _w}) = \textsf{obs}_{\textsf{input}}({\pi _c}) = 1, 2$.

In order to define a wider class of Heisenbugs that takes into account that the duration of quiescence may be either outside the control of the user or induced by the system, we give a new definition of input equality modulo quiescence.

### Definition 14

Let $(X, I, \textsf{init}, \textsf{final}, T)$ be an STS with a traces $\pi , \pi '$ and J⊆I a subset of inputs. Then the equality of input sequences modulo quiescence is defined as follows:

$$\begin{aligned} ({J}({\pi }) =_{\negmedspace /\tau }{J}({\pi '})) {\mathop {=}\limits ^{\tiny def }}\bigwedge _{i \in J} (\textsf{obs}_{i}({\pi }) = \textsf{obs}_{i}({\pi '})) \end{aligned}$$

Modifying Definition 7 to use this new notion of input equality allows us to identify Heisenbugs even if they arise from different input timings:

### Definition 15

An STS $(X, I, \textsf{init}, \textsf{final}, T)$ contains a Heisenbug *potentially induced by quiescence* with respect to an assertion φ if

$$\begin{aligned} \exists \pi _c, \pi _w\, .\, {I}({\pi _c})=_{\negmedspace /\tau }{I}({\pi _w})\wedge \pi _c \not \models \varphi \wedge \pi _w \models \varphi . \end{aligned}$$

The execution $\pi _c$ is the *counterexample execution*, $\pi _w$ is the *witness execution*.

### Example 18

The program in Example 16 has a Heisenbug with counterexample execution $\pi _c$ and witness execution $\pi _w$.

### Quiescence and causality

The arrival time of inputs and periods of quiescence can be viewed as an additional source of nondeterminism. Analogously to the determinization of an STS (Definition 9) where mechanisms are made explicit via refinement (Definition 8), this type of nondeterminism can be addressed by making quiescent inputs observable:

#### Definition 16

(Nonquiescent Determinization) Given some STS $S{\mathop {=}\limits ^{\tiny def }}(X, I, \textsf{init}, \textsf{final}, T)$, a *nonquiescent determinization* of *S* is a determinization ${S}_{\textsf{det}} {\mathop {=}\limits ^{\tiny def }}(X \uplus {X}_{\textsf{det}}, I \uplus M, {\textsf{init}}_{\textsf{det}}, {\textsf{final}}_{\textsf{det}}, {T}_{\textsf{det}})$ of *S* where values of the mechanisms m∈M range over $\textsf{Val} \setminus \{\tau \}$ and

2 $$\begin{aligned} \forall \pi , \pi '. \left( {I}({\pi }) =_{\negmedspace /\tau }{I}({\pi '}) \wedge {I}({\pi }) \not = {I}({\pi '})\right) \Rightarrow ({M}({\pi }) \not = {M}({\pi '}))\nonumber \\ \end{aligned}$$

holds.

Intuitively, Equation 2 in Definition 16 states that any discrepancy between the observable input sequence (Definition 13) and the actual input sequence (which may have periods of quiescence) must be made explicit in the refinement.

#### Proposition 3

Let $S{\mathop {=}\limits ^{\tiny def }}(X, I, \textsf{init}, \textsf{final}, T)$ be an STS. Then, a nonquiescent determinization ${S}_{\textsf{det}} {\mathop {=}\limits ^{\tiny def }}(X, I \uplus M, {\textsf{init}}_{\textsf{det}}, \textsf{final}, {T}_{\textsf{det}})$ of *S* always exists.

#### Proof

As in Proposition 2, we can obtain a deterministic refinement ${S}_{\textsf{det}} = (X, I \uplus {I}_{\textsf{det}}, {\textsf{init}}_{\textsf{det}}, \textsf{final}, {T}_{\textsf{det}})$ of *S* by adding an oracle variable $\textsf{oracle}$ and we have ${I}_{\textsf{det}} = {\textsf{oracle}}$; we recall that the values of $\textsf{oracle}$ fix the successor state, in particular, the value domain of $\textsf{oracle}$ can be chosen as set of all states (which do not contain the value τ). In addition, in order to ensure a nonquiescent determinization, we set $M = {I}_{\textsf{ref}} \uplus \{\textsf{i}_{\tau } | \textsf{i} \in I\}$ where for all $\textsf{i} \in I$, the values of the boolean variables $\textsf{i}_{\tau }$ indicate whether the value of $\textsf{i}$ is quiescent, i.e., the value of $\textsf{i}_{\tau }$ is $\textsf{true}$, if the value of $\textsf{i}$ is τ, and $\textsf{false}$, otherwise. By this construction, the mechanism will always differ if there is a discrepancy in the timing of quiescent values, thus ensuring nonquiescence. Finally, set ${\textsf{init}}_{\textsf{det}} = {\textsf{init}}_{\textsf{ref}}$ and ${T}_{\textsf{det}} = {T}_{\textsf{ref}}$. □

#### Example 19

In order to construct a nonquiescent deterministic STS for the program in Example 16, add a set of mechanisms $M = \{\textsf{thread}, \textsf{i}_{\tau }\}$ where $\textsf{thread}$ models the thread picked by the scheduler (similar to Example 8) and $\textsf{i}_{\tau }$ indicates quiescence in the input as described in the proof of Proposition 3.

Nonquiescent determinizations allow us to identify causes of a nonquiescent Heisenbug similar to Definition 10.

#### Definition 17

Let $S{\mathop {=}\limits ^{\tiny def }}(X, I, \textsf{init}, \textsf{final}, T)$ be a nondeterministic STS with a Heisenbug (potentially induced by quiescence, as in Definition 15) with respect to an assertion φ, and let ${S}_{\textsf{det}} {\mathop {=}\limits ^{\tiny def }}(X \uplus {X}_{\textsf{det}}, I \uplus M, {\textsf{init}}_{\textsf{det}}, {\textsf{final}}_{\textsf{det}}, {T}_{\textsf{det}})$ be a nonquiescent determinization of *S*. Let $M=M_C\uplus M_N$. We say that $M_C$ is a *cause* with respect to *M* and φ iff

3 $$\begin{aligned} \exists \pi _c, \pi _w .\, {I}({\pi _c})&=_{\negmedspace /\tau }&{I}({\pi _w}) \wedge \nonumber \\ {M_N}({\pi _c})= &   {M_N}({\pi _w}) \wedge \pi _c \not \models \varphi \wedge \pi _w \models \varphi \end{aligned}$$

and $M_C$ is a minimal subset of *M* with this property.

Note that Equation 3 in Definition 17 mirrors Equation 1 in Definition 10.

#### Proposition 4

(Existence of a Cause under Quiescence) Given a nondeterministic STS $S{\mathop {=}\limits ^{\tiny def }}(X, I, \textsf{init}, \textsf{final}, T)$ with a Heisenbug potentially induced by quiescence (Definition 15) with respect to an assertion φ, let ${S}_{\textsf{det}} {\mathop {=}\limits ^{\tiny def }}(X \uplus {X}_{\textsf{det}}, I \uplus M, {\textsf{init}}_{\textsf{det}}, {\textsf{final}}_{\textsf{det}}, {T}_{\textsf{det}})$ be a nonquiescent determinization of *S*. Then there exists a nonempty cause $M_C$ with respect to *M* and φ.

#### Proof

Let $\pi _c$ and $\pi _w$ be executions of *S* that satisfy Definition 15, i.e., ${I}({\pi _c})=_{\negmedspace /\tau }{I}({\pi _w})$, $\pi _c \not \models \varphi$, and $\pi _w \models \varphi$. As in the proof of Proposition 1, let ${\pi _c}_{\textsf{det}}$ and ${\pi _w}_{\textsf{det}}$ be the corresponding traces in ${S}_{\textsf{det}}$ and let $M_N \subseteq M$ such that $m \in M_N$ if and only if ${m}({{\pi _c}_{\textsf{det}}}) = {m}({{\pi _w}_{\textsf{det}}})$ and $M_C = M \setminus M_N$. If ${I}({\pi _c}) = {I}({\pi _w})$, the proof is similar to Proposition 1. Otherwise, since ${S}_{\textsf{det}}$ is nonquiescent, ${M}({\pi _c}) \not = {M}({\pi _w})$. However, this means that there must be at least one $m \in M_C$, i.e., $M_C$ is nonempty. □

#### Example 20

The traces $\pi _c$ and $\pi _w$ in Example 16 show that there is a Heisenbug with cause $\textsf{i}_{\tau }$ (cf. Example 19). Note that the mechanism $\textsf{thread}$ is also a cause in this example, as it is also possible to find two traces that only differ in the schedule, but have different results with respect to the assertion.

## A taxonomy of potential causes

The results in Sect. 3.2 imply that the accuracy of any causal analysis is contingent on the set of mechanisms considered as potential causes. To provide a better understanding of potential causes of Heisenbugs and aid (manual or automated) causal analyses, we list representative mechanisms that can underlie Heisenbugs and provide a systematic classification. Additionally, we identify cross-cutting patterns occurring in various categories. Based on the categorization, we discuss practical concerns regarding observability and controllability of the mechanisms.

### Categories of causes

Every mechanism belongs to one class according to the following two schemes.

#### Nondeterminism and system alterations

We distinguish two fundamentally different classes of mechanisms: inherent nondeterminism in the system $\left( \text {indicated by~}{{\boldsymbol{?}}}\right)$ and alterations which results in different versions of the system (indicated by ). Note that some nondeterministic systems can be altered in a way that reduces nondeterminism and hence possibly eliminates Heisenbugs. Peterson’s algorithm (Example 3), for instance, is correct on platforms that guarantee sequential consistency, as memory accesses will not be reordered. Consequently, weak memory models give rise to mechanisms in both classes. Below, we assign such examples to ***?***, as the original nondeterministic program already contains a Heisenbug independently of the alteration. Section 3 revealed that the alteration is not causally related to the Heisenbug in such cases.

#### System level

Causes of Heisenbugs can be located at different system levels. We distinguish the following levels (closely following [51]):

➀: Digital logic level
➁: Microarchitecture level
➂: Instruction set architecture level
➃: Operating system level
➄: Compiler level
➅: High-level program level

### Cross-cutting patterns

The following patterns occur across different categories and system levels:

- **Concurrency** ⇊Whenever several concurrent (partially ordered) events are linearly (i.e., totally) ordered, there is a number of linear extensions, each of which corresponds to a possible schedule. The choice of such a schedule is (or may seem) nondeterministic.
- **Timing** The current time (or a time span) might—if not considered as an explicit system input—influence the outcome, resulting in nondeterminism.
- **Platform** ❏ The specific characteristics of a mechanism may depend on the underlying platform (e.g., processor architecture or software stack).

### Classification of example causes

The following examples are organized by the system level they occur on and labeled with the tags of the categories they belong to.

**Table:** 

**Hardware faults**	➀***?***
Manufacturing errors, aging, environmental conditions occasionally cause transient or intermittent faults [56], such as bit flips in memory or storage.	
**Processor clock drift**	➀***?***
Clocks of different components run at different speeds and may drift apart after synchronization. The time since last synchronization is nondeterministic.	
**Weak Memory Models**	➁***?*** ⇊❏
On multiprocessor platforms, micro-architectural features (e.g., caches) can delay propagation of writes to shared memory, yielding different orderings in which concurrent memory accesses are observed across cores [2] (cf. Example 3).	
**CPU instruction reordering**	➁***?*** ⇊
Pipelined CPUs execute independent instructions out of order. Reordering of shared memory accesses is visible to parallel processes [21].	
**Byte ordering**	➂ ❏
Different processor architectures store bytes in opposite orders, with the most significant byte first (big endian) or the least significant byte first (little endian) [51], yielding deviating results for programs accessing individual bytes.	
**Word and register size**	➂ ❏
Sizes of registers and memory cells vary across architectures, yielding different overflow behavior, e.g., 32- vs 64-bit integers, or Intel x87 80-bit floating point registers and 64-bit floating point memory cells [39] (see Example 2).	
**Scheduling**	➃***?*** ⇊
Different interleavings of thread or process execution lead to deviating results, e.g., in the context of race conditions or atomicity violations [37] (see Example 1).	
**Network package reordering**	➃***?*** ⇊
Network packets may be received in a different order than they were sent, e.g., because of network routing or parallel processing in network devices [6].	
**Network package loss**	➃***?***
Network packets may be dropped due to congestion or transmission errors. For protocols such as UDP, loss is visible to the application [30].	
**Data structure alignment**	➄
For performance reasons, compilers may align the addresses of data structures to coincide with multiples of the architecture’s word size (e.g., 32- or 64-bit). Smaller fields may be padded to conform with this alignment; denser alignments can be forced using the-fpack-struct flag [47]. This may lead to different outcomes when fields are directly accessed by address.	
**Undefined behavior**	➄ / ***?***
Programming constructs whose behavior is underspecified in the language standard can have different semantics depending on compiler and optimization level [28, Section J.2], e.g., in case of integer overflows, uninitialized memory, or race conditions. The latter two, for instance, can result in nondeterminism.	
**Debugging statements**	➅
The insertion of code for debugging purposes may introduce a probe effect, influencing data-flow (see Example 2) or control-flow (see Example 3).	
**Disabling assertions**	➅
Disabling assertions for production code, e.g., using the C macro NDEBUG [28, Section 7.2] may change program behavior due to side effects even if all assertions are satisfied.	
**Pseudo-random numbers**	➅***?***
Pseudo-random number generators can produce different sequences of random numbers in every execution, unless a seed is set, see, e.g., [29].	
**Time-dependency**	➅***?***
Time can be an implicit parameter changing application logic, see, e.g., leap year bugs or a bug occurring only on Wednesdays [43].	
**Library versions**	➅ ❏
Different library/component versions or implementations might deviate in behavior, cf. a bug caused by different versions of Java HashMap [52].	

### Observability and controllability

Observing and controlling nondeterministic mechanisms—the analogon to refinement in Sect. 3.1—represents the main obstacle for a causal analysis of Heisenbugs. Regarding observability and controllability, actual nondeterminism and system alterations have fundamentally different properties. System alterations often constitute the easier case, as the task is to observe and control which system version is currently used, which corresponds to observing or modifying one initial decision. In case of hardware related mechanisms such as the register size, observing the decision amounts to checking which processor version is used and controlling it amounts to using a different processor. Similarly, for software related mechanisms such as configurations with respect to compilation or logging settings, the task is to observe or swap the configuration.

Inherent nondeterminism (such as concurrency) usually yields a sequence of nondeterministic decisions. Whether it is possible to observe and control these decisions highly depends on the execution environment. In many cases the decisions can only be made observable and controllable using some kind of instrumentation of code or hardware. In software this can be readily achieved by means of logging. Observability is typically more restricted in hardware as the chip cannot be simply stopped to inspect all on-chip signals; hence, observability in hardware depends on the availability of mechanisms such as trace buffers, scan chains, or hardware performance counters. Moreover, instrumentation itself constitutes a system alteration, which might introduce a probe effect (especially in systems which are sensitive to timing or the order of concurrent events).

Both problems can be mitigated by relying on virtualization, simulation, or the (potentially automated) analysis of a formal model of the system. While these techniques enable a step-by-step inspection or modification of the system, the validity of the results is contingent on the faithfulness of the virtualization environment, simulator, or model, i.e., the execution model of the simulator or virtualization must not deviate from the original system.

## Analysis methodology and challenges

We sketch an (iterative) methodology for practical analyses based on the formalization above and showcase two possible instantiations and their challenges:

- ➀ **Task:** Starting from a Heisenbug (Definition 7), identify candidate mechanisms *M* (e.g., consulting the taxonomy in Sect. 5 or surveys [42]).
  **Challenge:** The accuracy of the analysis is contingent on identifying the relevant mechanisms.
- ➁ **Task:** Pick a mechanism $\textsf{m}\in M$ and adapt (or refine according to Definition 8) the model or system to make m controllable (or at least observable).
  **Challenge:** The system may be inherently uncontrollable or unobservable, or attempts to control/observe it potentially introduce a probe effect.
- ➂ **Task:** Identify (contributing) causes by finding witnesses that deviate in as few mechanisms as possible (i.e., satisfy Equation 1 in Definition 10).
  **Challenge:** Testing will yield overapproximations only (cf. Proposition 1).
- ➃ **Task:** Check a stopping criterion to determine whether further mechanisms or refinement steps are required (steps ➀ and ➁).
  **Challenge:** Assessing whether all causes have been correctly identified is challenging and may amount to fixing the bug and re-verifying the system.

### Causal analysis based on model checking

We built a NuSMV [9] model of Peterson’s algorithm (Listing 1.3). (The source code of the model is provided in Sect. A.) We use self-composition [4], which composes two copies $S_w$ and $S_c$ of the STS *S*, to reduce the existence of a counterexample trace and a witness trace (which is a hyperproperty) to the existence of a single trace in the composed model.^4^ NuSMV can then construct the trace as a counterexample to an LTL property over the composed model. As NuSMV usually considers infinite traces, final conditions are accounted for in the property. The existence of a Heisenbug can be confirmed by checking that NuSMV finds a counterexample to the property $\psi := G (\textsf{final} _c \wedge \textsf{final} _w \Rightarrow (\varphi _w \Rightarrow \varphi _c))$ for $\textsf{final}$ and φ as in Example 10 and Example 11 (where subscripted predicates range over the matching variable set).

In step ➀, we pick the fact whether the print statements are executed and model it adding variables $\textsf{print}_w$ and $\textsf{print}_c$ to the model (step ➁). In step ➂, we invoke NuSMV on the property $G (\textsf{print}_w \Leftrightarrow \textsf{print} _c) \Rightarrow \psi$. As there is a counterexample, we identify the empty set as a contributing cause.

We start another refinement iteration, pick concurrency as mechanism (step ➀) and model it by variables $\textsf{thread}_w$ and $\textsf{thread}_c$ (step ➁). We check the property $G ((\textsf{print}_w \Leftrightarrow \textsf{print} _c) \wedge (\textsf{thread}_w \Leftrightarrow \textsf{thread}_c)) \Rightarrow \psi$ (step ➂). Again, there is a counterexample and the empty set is a contributing cause.

In the next refinement iteration, we pick the weak memory behavior (step ➀) we model it by variables $\textsf{delay}_w$ and $\textsf{delay}_c$ and reflect the fact that $\textsf{print} \implies \lnot \textsf{delay}$ (cf. Example 10) (step ➁). Checking property $G ((\textsf{print}_w \Leftrightarrow \textsf{print}_c) \wedge (\textsf{thread}_w \Leftrightarrow \textsf{thread}_c) \wedge (\textsf{delay}_w \Leftrightarrow \textsf{delay}_c)) \Rightarrow \psi$ returns true, hence we have now found a nonempty cause superset and can start cause minimization. A counterexample to $G ((\textsf{print}_w \Leftrightarrow \textsf{print}_c) \wedge (\textsf{thread}_w \Leftrightarrow \textsf{thread}_c)) \Rightarrow \psi$ witnesses that $\textsf{delay}$ is a cause, similarly a counterexample to $G ((\textsf{print}_w \Leftrightarrow \textsf{print}_c) \wedge (\textsf{delay}_w \Leftrightarrow \textsf{delay}_c)) \Rightarrow \psi$ witnesses that $\textsf{thread}$ is a cause. As the model satisfies $G ((\textsf{delay}_w \Leftrightarrow \textsf{delay}_c) \wedge (\textsf{thread}_w \Leftrightarrow \textsf{thread}_c)) \Rightarrow \psi$, $\textsf{print}$ is not a cause. This concludes step ➂. As we identified a nonempty cause, no more refinement steps are needed.

### Test-based causal analysis

**Figure:**
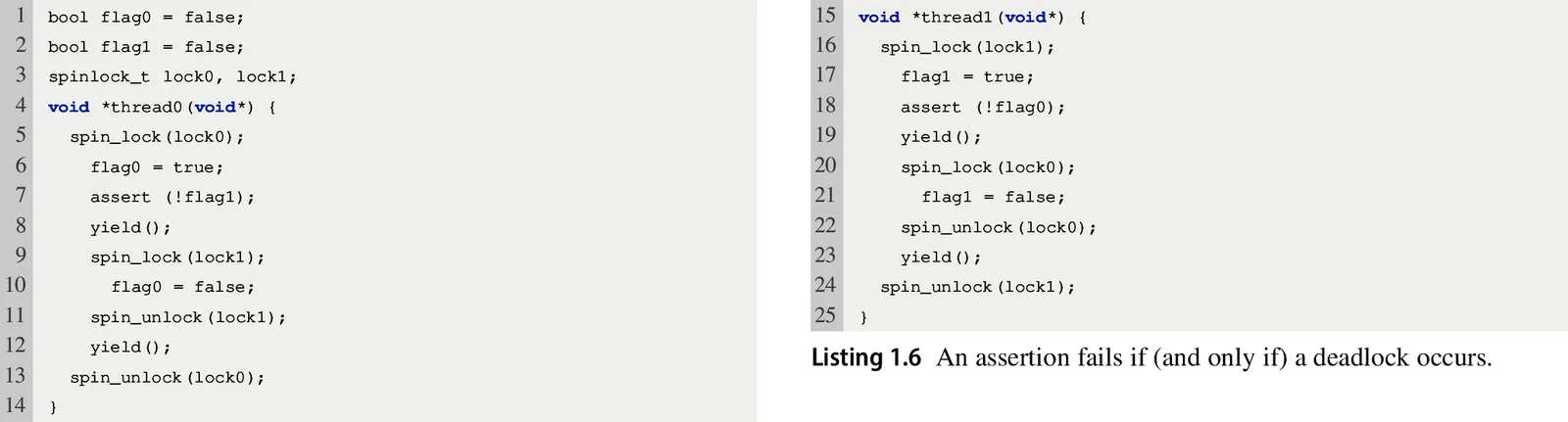

Consider the code in Listing 1.6, which might deadlock because of a faulty locking discipline. The assertions in lines 7 and 18 fail when a deadlock, caused by a specific (combination of) context switche(s), occurs: a context switch at line 8 to thread1 (or, symmetrically, from line 19 to thread0) causes both threads to wait for a lock held by the other thread.

In step ➀, we identify concurrency (limited to the context switches marked by yield for simplicity) as potential cause. Following the approach of KISS [44], we control the scheduler (step ➁) by sequentializing the concurrent program and simulating the execution of a large subset of its interleavings. See the paragraph *Sequentialization Approach* below for a discussion of the technical details. In KISS, threads can be started and terminated nondeterministically at any point during the execution. Using closures to save the local state of a thread, we add the capability to *re-enter* a thread after its interruption by yield. The execution of thread0 (thread1, respectively) can be interrupted at lines 8 and 12 (19 and 23, respectively). Our sequentialization enables us to explicitly control these four context switches, inducing $2^4$ potential schedules. Random (or systematic) exploration of these schedules then yields executions that terminate normally or violate an assertion. Failing executions deviate from the nonfailing ones by performing a context switch at lines 8 or 19, at least one of which must constitute (part of) the candidate cause(s) we identify in step ➂.

Testing merely provides an overapproximation of the cause $M_C$ (Proposition 1). Due to the minimality requirement in Definition 10 and Definition 11, however, removing one element from $M_C$ (by controlling the mechanism accordingly) eliminates the entire cause. Assume for now, that thread0 in Listing 1.6 always executes first, in which case the context switch at line 8 is a unique cause for the deadlock. Consider an overapproximation comprising of two context switches at lines 8 and 19. Blocking the context switch at line 8 eliminates the Heisenbug, while blocking the one at line 19 doesn’t. By individually blocking the context switches and checking whether subsequent testing provides sufficient confidence that the bug has been eliminated, we obtain a stopping criterion in step ➃.

If, however, executions may start with thread0 or thread1, the context switches at lines 8 and 19 form two independent (nonintersecting) causes (due to the symmetry in Listing 1.6). Consequently, *both* context switches must be identified to eliminate all causes of the bug (cf. Sect. 3.4). Blocking individual context switches (as suggested above) does not provide a reliable stopping criterion. Despite this limitation, testing-based analysis can help the developer to narrow down the set of candidate causes significantly.

#### Sequentialization approach

As stated above, we perform a sequentialization of Listing 1.6 in the spirit of KISS [44].^5^ Unlike KISS, however, our sequentialization exploits C++ closures to allow threads to be re-entered after they are interrupted by yield.^6^

**Figure:**
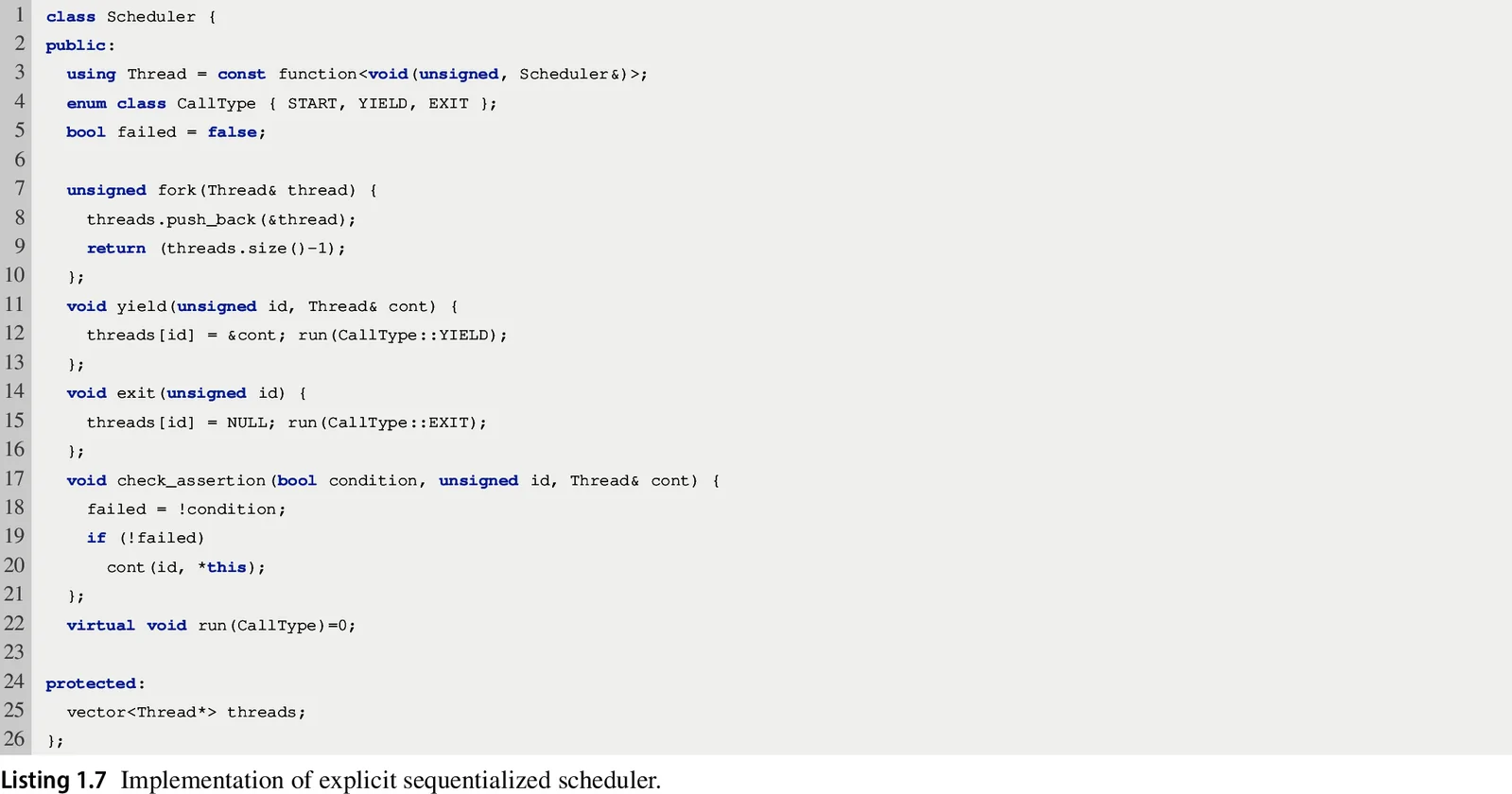

Listing 1.7 shows the implementation of our explicit and sequentialized scheduler. The methods fork, yield and check_assertion take a Thread function (whose type is declared in line 3), which the calling thread uses to provide the remaining code to be executed after the call to the scheduler returns. The virtual function run implements the scheduling policy.

**Figure:**
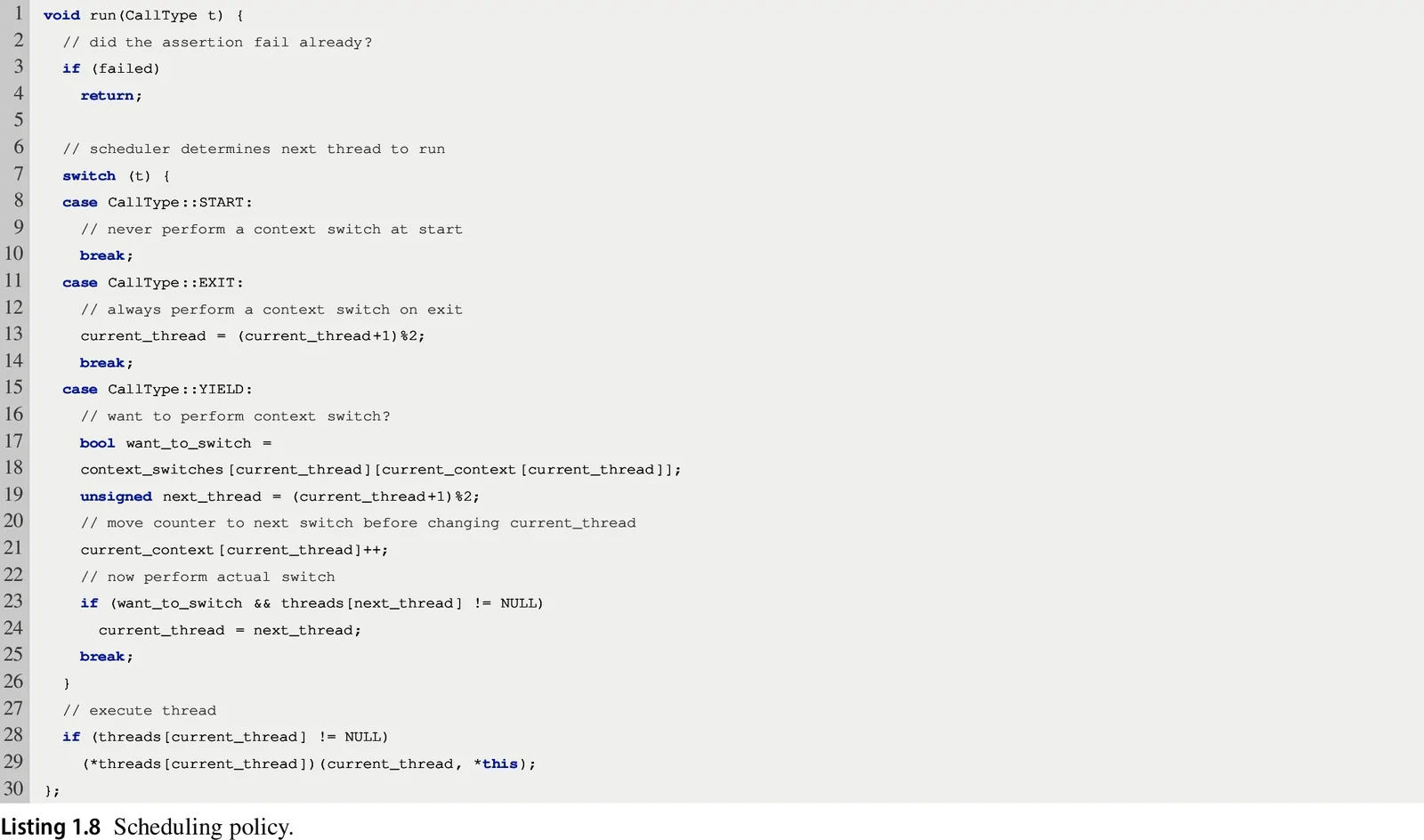

Listing 1.8 shows a potential deterministic scheduling policy for the two threads in Listing 1.6. In line 15, run consults the array context_switches to determine whether the current thread (current_thread) should perform a context switch in the current context (current_context[current_thread]) (i.e., context_switch constitutes a prophecy variable). This array can be generated randomly or systematically (e.g., by generating all $2^4$ possibilities, or by systematically mutating an initial schedule).

**Figure:**
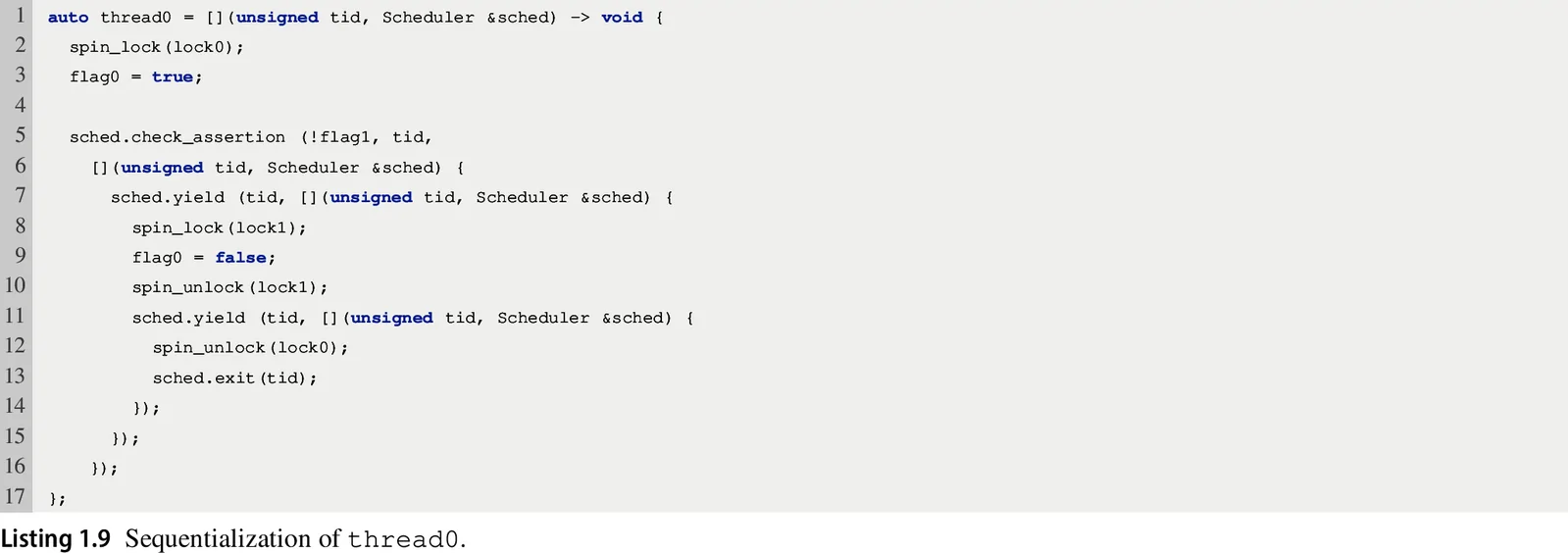

Finally, Listing 1.9 shows the sequentialization of thread0, using lambda functions/closures to pass the remaining code to be executed to the methods of the scheduler.

**Fig. 7:**
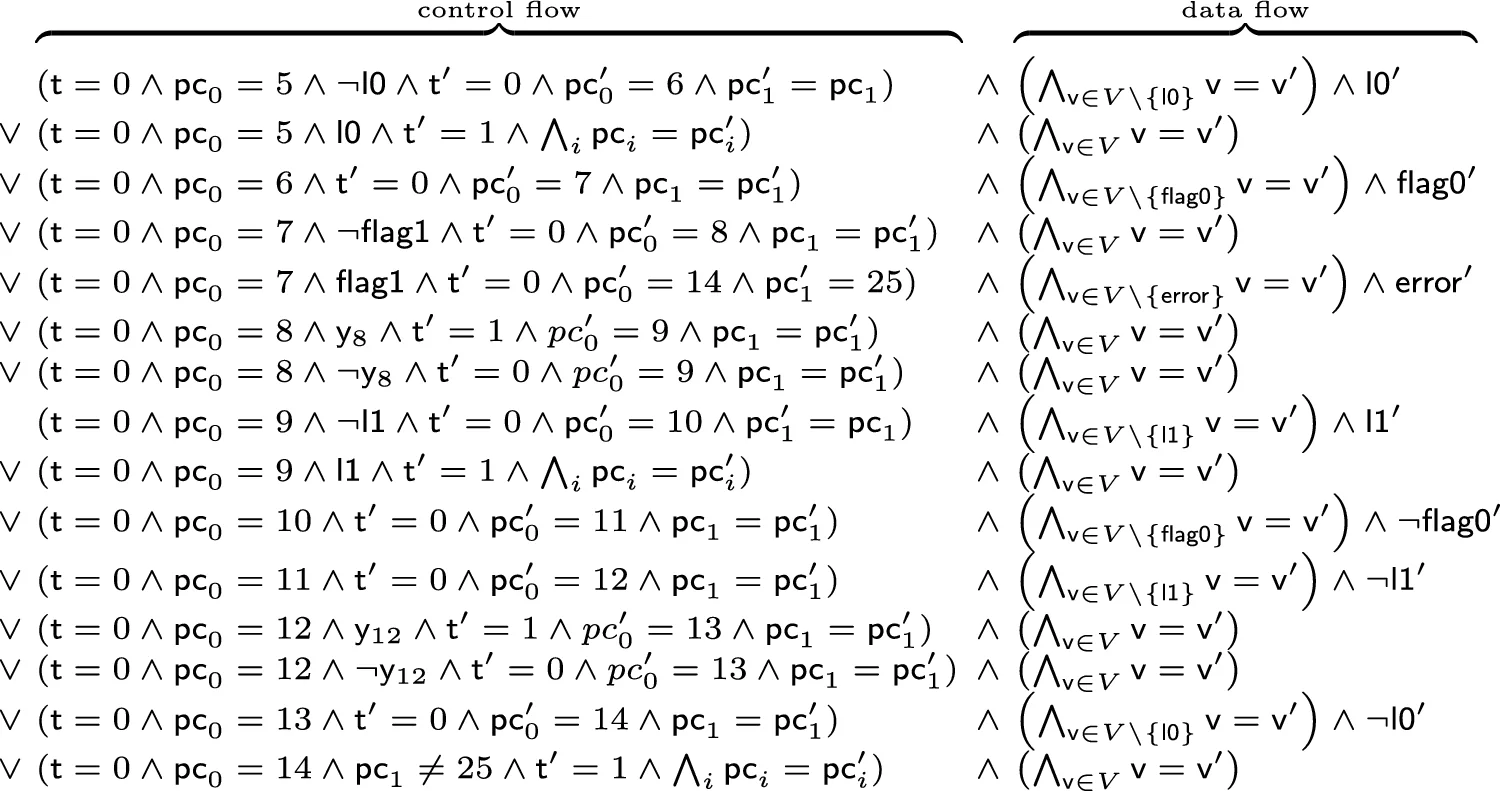
Encoding of Listing 1.6

#### Refinement and transition relation

For the sake of completeness, we provide the encoding of the transition relation of thread0 (thread1 is symmetric) of Sect. 6.2, which is shown in Fig. 7. Here, the assertions are represented by a variable $\textsf{error}$ which is initialized to false. As the code in Listing 1.6 features two assertions, a failing assertion transitions into a dedicated final state specified by $(\mathsf {pc_0}=14\wedge \textsf{pc}_1=25)$. The variable $\textsf{t}$ indicates the current thread and $\textsf{y}_{\ell }$ indicates whether there’s a context switch at program location $\ell \in \{8,12,19,23\}.$,^7^ Hence, $I=\{\textsf{t}, \textsf{y}_8, \textsf{y}_{12}, \textsf{y}_{{19}}, \textsf{y}_{23}\}$ represents the set of potential causes of the deadlock.

To identify causes (Definition 10), we need to identify two minimally deviating executions. Consider the executions with the following initial conditions:

$$\begin{aligned} \begin{array}{lclcrclclcl} \textsf{init} _1& \equiv & (\textsf{t}=0) & \wedge &  \textsf{y}_8 &  \wedge &  \lnot \textsf{y}_{12} & \wedge &  \lnot \textsf{y}_{19} &  \wedge &  \lnot \textsf{y}_{23} \\ \textsf{init} _2& \equiv & (\textsf{t}=0) & \wedge &  \lnot \textsf{y}_8 &  \wedge &  \lnot \textsf{y}_{12} & \wedge &  \lnot \textsf{y}_{19} &  \wedge &  \lnot \textsf{y}_{23} \\ \end{array} \end{aligned}$$

The condition $\textsf{init} _1$ leads to a deadlock, whereas $\textsf{init} _2$ results in a sequentialized execution of thread0 and thread1. The values of the variables in *I* only deviate for $\textsf{y}_8$, hence the context switch at line 8 constitutes a cause for the deadlock. Symmetrically, the context switch at line 8 also causes a deadlock.

Not all context switches constitute causes, though, as executions satisfying $(\lnot \textsf{y}_8\wedge \lnot \textsf{y}_{19})$ do not lead to deadlocks independently of the values of the other variables in *I*.

## Related work

*Terminology and Definition of Heisenbugs* The first paper mentioning Heisenbugs [24] uses the term for transient software bugs which disappear under observation. In [25], bugs are classified into Bohrbugs (bugs manifesting consistently), Mandelbugs (bugs with complex error propagation), and Heisenbugs (bugs manifesting differently under the probe effect). In contrast to this informal classification, our definition is formal, covering Heisenbugs which stem from the probe effect as well as from nondeterminism. The term is frequently (and informally) used in the context of concurrency [41], where it exclusively refers to bugs caused by control-flow nondeterminism. In the context of testing, the notion of flaky tests [42] resembles the notion of Heisenbugs. The comparison of failing and nonfailing executions is used in several lines of research, the goals of which are orthogonal to the definition of bug classes. Differential assertion checking [31] compares failing and nonfailing executions to define relative correctness of different program versions.

[10] introduces the notion of critical pairs, a pair of one failing and one nonfailing execution which are indistinguishable with respect to certain observations, in order to check diagnosability of system faults.

*Causality* Our definition of causality is inspired by Lewis’ counterfactuals [35]. The negation of Definition 10 mirrors the definition of causal irrelevance in [22] and Definition 10 corresponds to its dual notion of causality between variables [16]. A core difference is that our interventions are restricted to inputs that represent nondeterministic mechanisms rather than affecting arbitrary points of the transition relation (or the causal model). Moreover, causal models have a fixed propagation depth, while we consider an arbitrary number of unwindings of the transition relation. Halpern and Pearl [26, 27] provide a widely accepted definition of “actual” causes based on counterfactuals, where contingencies are used to control interference between interventions. We provide a more detailed comparison of our work to actual causality in Sect. 7.1.

Several lines of work reason about the origin of system faults [3, 5, 14, 33] using Halpern and Pearl’s notion of causality. Gößler and Stefani [23] provide operators that build counterfactual executions, and support nondeterminism in systems as well as hyperproperties. In [12], actual causality is used to explain violations of hyperproperties. It formalizes causes for violations of (arbitrary) universally quantified hyperproperties as a hyperproperty with quantifier alternation, which can then be checked with a model checker such as [20]. We formalize causes for Heisenbugs (a specific hyperproperty) in terms of an existentially quantified hyperproperty and provide criteria under which only singleton sets have to be considered as causes.

Several approaches exist for automatically detecting causes of flaky tests. The RootFinder tool [32] collects passing and failing executions and correlates their differences with a specific cause. In [57] the authors present a tool for finding code locations that lead to differences between succeeding and failing executions. Identifying what happens in these locations is left to the developer. In [53] and [40] the system is repeatedly executed under different configurations to check which configuration influences the manifestation of the bug. All of these approaches are based on computing correlations rather than performing rigorous causal inference. In contrast, our framework is based on a formal causal analysis accounting for interactions of multiple potential causes.

*Surveys and Taxonomies* [42] provides a taxonomy of causes relevant in the context of automated testing. In [13], Mandelbugs are categorized according to whether their cause is related to time lags, the system environment, the timing, or the relative order of operations. Furthermore, there are several papers investigating why reported bugs are not reproducible [17, 45]. Among other reasons (such as missing information in the bug report), they name different execution environments and nondeterminism in the program, but do not differentiate specific mechanisms. [37] provides a comprehensive survey of concurrency bugs.

### Relationship to actual causality

In this section, we discuss the relationship of our notion of causality (introduced in Sect. 3) and the widely used definition of *actual causality* introduced by Halpern and Pearl [26, 27] (see Sect. 7.1.1).

#### Actual and general causes

Halpern and Pearl’s work on *actual causes* [26, 27] is a prominent attempt to formalize causality in *specific* scenarios. Their framework relies on *structural equations* over variables to characterize assumptions about a given world and its causal relations. Specific assignments to variables represent primitive events, and structural equations (Boolean combinations of primitive events) determine what happens in case of interventions to the system. This can be illustrated by the canonical rock-throwing example (see, e.g., [26, Example 3.2]). Consider the following causal model4Here, $\textsf{st}$ and $\textsf{bt}$ indicate that Suzie (or Billy, respectively) throw stones at a bottle. The structural Equation 4 above encodes the following rules:

➀: If either Suzie’s or Billy’s stone hits the bottle, then the bottle shatters ($\textsf{bs}$).
➁: Suzie always throws first; hence, if Suzie throws her stone ($\textsf{st}$), she hits the bottle ($\textsf{sh}$).
➂: Billy’s stone throw hits the bottle ($\textsf{bh}$) unless Suzie already threw her stone.

The question is which event ($\textsf{st}$ or $\textsf{bt}$) is the cause for $\textsf{bs}$. Actual causality [26, 27] determines causality with respect to a specific scenario, e.g., the setting $(\textsf{st}\wedge \textsf{bt})$. In this scenario, it is undesirable to classify $\textsf{bt}$ as a cause, as Billy’s stone didn’t hit the bottle. Halpern and Pearl achieve this by introducing *contingencies* that prevent that the alternative scenarios that are considered deviate too much from the given reality (in this example, $\textsf{bh}$ will be fixed to false even if Suzie does not throw her stone; hence changing $\textsf{st}$ to $\lnot \textsf{st}$ will change the outcome $\textsf{bs}$).

Now, Equation 4 can be viewed as single-step transition relation. Unlike [26, 27], Definition 10 does not allow the modification of $\textsf{bh}$, as it is deterministically determined by $\textsf{st}$ and $\textsf{bt}$. Notably, in [26, 27], the introduction of variables such as $\textsf{bh}$ that model the causal process are crucial to prevent $\textsf{bt}$ from being considered a cause. Definition 10 treats the transition system as a black box, leaving no room for contingencies. Consequently, were we to misuse Definition 10 by fixing a reference trace with $(\textsf{st}\wedge \textsf{bt})$, $\textsf{bt}$ would still be considered a cause.

Unlike in Halpern and Pearl’s framework, our formalization of a cause is not relative to a specific reference execution and hence closer to a general (or *type-level*) cause. Our formalization (Definition 10) will consider *all* scenarios (i.e., combinations of values for $\textsf{st}$ or $\textsf{bt}$) and hence imply that both $\textsf{st}$ and $\textsf{bt}$ are potential causes.

Notably, the framework by Halpern and Pearl has been modified and improved over the years. Because of an idiosyncrasy of Halpern and Pearl’s framework regarding the number of events in their causes, causes in their *original* model of causality [27] are *always* singleton sets [16]. In [26], causes are conjunctions of events if the individual events cannot *independently* change the outcome (where independently is to be interpreted as in the context of Modified Condition/Decision Coverage).

Similarly to [26], our notion of causality allows causes to be sets of events/mechanisms, but the underlying reasons for this phenomenon are subtly different: in Sects. 3.5 and 3.6, we analyze under which conditions causes in our setting comprise a single mechanism only.

## Conclusion

While the term Heisenbug is widely used, its exact meaning often depends on the context. We provide a formal definition that unifies the notion of Heisenbugs caused by a system alteration and those caused by nondeterminism. Furthermore, we present a hyperproperty-based framework for determining which mechanisms cause the manifestation of a Heisenbug. In particular, our approach allows the identification of causes in the presence of multiple mechanisms that could trigger a Heisenbug and gives guarantees for results of a causal analysis even in presence of nondeterminism. Building on this result, we sketch a methodology for causal analysis based on iterative refinement, which can be informed by our taxonomy of potential causes. Moreover, we provide a detailed comparison of our approach with Halpern and Pear’s actual causes.

Our work facilitates future development of systematic debugging methodologies and automated tools for identifying Heisenbugs and their causes. As a first step in this direction, we plan to compile a benchmark suite of examples for all presented mechanisms, which can be used for evaluation.

## Auxiliary material for Peterson’s algorithm

See Listing 1.10 for the NuSMV model of Peterson’s algorithm (Listing 1.3).

## Footnotes

1 In the literature, sometimes the term Mandelbug is used as an umbrella term for the mentioned categories. However, Mandelbugs additionally include complex faults where there is “a delay between the fault activation and the final failure occurrence”.

2 We assume that ∧ has precedence over ∨.

3 As pointed out by a reviewer, the assumption that any violation of an assertion amounts to a bug is a design decision, as specifications may be faulty, too, or the programmer may have misunderstood the semantics of the execution model. We discuss this matter in more detail in [8], where we present a novel technique to iteratively refine inadequate specifications.

4 While tools that check hypersafety properties directly (such as McHyper [20] or AutoHyper [7]) are available, they also essentially apply self-composition or product construction [4]—which is why we decided to illustrate this approach explicitly.

5 While this sequentialization has been done manually, it can in principle be automated.

6 Alternatively, this could be achieved using the recently introduced C++ co-routines.

7 We limit the number of context switches to keep the encoding in Fig. 7 simple. Note that context switches can also be triggered by failed lock acquisition attempts (lines 9 and 20). Unlike with yield failed calls to spin_lock leave the program counters unchanged, potentially introducing nonterminating executions of the transition system. As that situation represents exactly the deadlock detected by the assertions, however, such failing acquisition attempts are effectively unreachable.
